# Highly Pathogenic Avian Influenza A(H7N9) Virus, Tennessee, USA, March 2017

**DOI:** 10.3201/eid2311.171013

**Published:** 2017-11

**Authors:** Dong-Hun Lee, Mia K. Torchetti, Mary Lea Killian, Yohannes Berhane, David E. Swayne

**Affiliations:** US Department of Agriculture, Athens, Georgia, USA (D.-H. Lee, D.E. Swayne);; US Department of Agriculture, Ames, Iowa, USA (M.K. Torchetti, M.L. Killian);; National Centre for Foreign Animal Disease, Winnipeg, Manitoba, Canada (Y. Berhane)

**Keywords:** highly pathogenic avian influenza virus, HPAI virus, H7N9, wild bird, poultry, mutation, phylogenetic analysis, Tennessee, Alabama, Wyoming, Iowa, United States, viruses, respiratory infections, zoonoses, transmission, influenza, farm, commercial farm, backyard farm, chicken

## Abstract

In March 2017, highly pathogenic avian influenza A(H7N9) was detected at 2 poultry farms in Tennessee, USA. Surveillance data and genetic analyses indicated multiple introductions of low pathogenicity avian influenza virus before mutation to high pathogenicity and interfarm transmission. Poultry surveillance should continue because low pathogenicity viruses circulate and spill over into commercial poultry.

In early March 2017, concurrent outbreaks of highly pathogenic avian influenza (HPAI) and low pathogenicity avian influenza (LPAI) A(H7N9) were occurring at poultry farms in Tennessee, USA. The first report of high loss due to death was received from a commercial broiler breeder facility in Lincoln County, Tennessee. The facility contained 74,000 chickens in 6 houses, but only 1 house was affected. Signs of disease included respiratory distress and increased death. Two days after disease onset, the number of dead birds increased from 50 to 500 within 24 hours, and oropharyngeal swab samples tested positive by real-time reverse transcription PCR (rRT-PCR) for the matrix and H7 genes at the C. E. Kord Animal Health Diagnostic Laboratory (Nashville, Tennessee). Samples were forwarded to the National Veterinary Services Laboratories of the US Department of Agriculture (Ames, Iowa, USA) for confirmation, and depopulation of the flock was initiated. The next day, the virus was confirmed as H7N9 HPAI virus by sequencing. A second HPAI-positive broiler breeder flock of 55,000 birds was identified in the control zone <10 days after identification of the first site; this site also had only 1 house affected. During the time between the 2 HPAI virus detections, H7N9 LPAI was confirmed in a different broiler breeder flock in Giles, a neighboring Tennessee county (Technical Appendix Figure 1). Subsequently, additional commercial and backyard flocks in Alabama, Kentucky, and Georgia were identified to have H7N9 LPAI through zone and routine surveillance. To trace the origin and understand their genetic relationship, we performed whole-genome sequencing and comparative phylogenetic analysis of the available H7N9 viruses identified in Tennessee and Alabama.

## The Study

We sequenced the complete genomes of 12 HPAI viruses (7 from the first and 5 from the second site), 3 LPAI viruses from Giles County, and 7 LPAI viruses from Alabama by next-generation sequencing (Technical Appendix Table). We generated maximum-likelihood phylogenies by using RAxML (*1*) and Bayesian relaxed clock phylogenies by using BEAST version 1.8.3 (*2*). After removing the insertion sequences at the hemagglutinin (HA) cleavage sites from HPAI viruses, we concatenated all available whole-genome sequences and analyzed them by using the median-joining method implemented by NETWORK version 5.0 (Technical Appendix) (*3*).

H7N9 HPAI and LPAI viruses from North America were genetically distinct from those from China, which have been infecting poultry and humans since 2013 (Technical Appendix Figure 2). All H7N9 viruses shared high levels of nucleotide identity (>99.2%–99.7%) across all 8 gene segments except for the insertion at the HA cleavage site. Insertion sequences, thought to be critical for the HPAI phenotype, elongate the proteolytic cleavage site, making HA more susceptible to cleavage by ubiquitous proteases. The insertion sequence at the HA cleavage site (PENPKTDRKSRHRRIR/G, insertion sequence is underlined) in H7 viruses had 100% sequence homology to chicken 28S rRNA (GenBank accession no. AC147447.3), suggesting the mutation occurred during virus replication in chickens. Similar events of chicken rRNA insertion have been reported in the North America H7N3 virus lineage in poultry (*4*). All previously reported H7 HPAI viruses had insertions near the HA cleavage site ranging from 6 to 54 nt (*5*).

**Figure 1:**
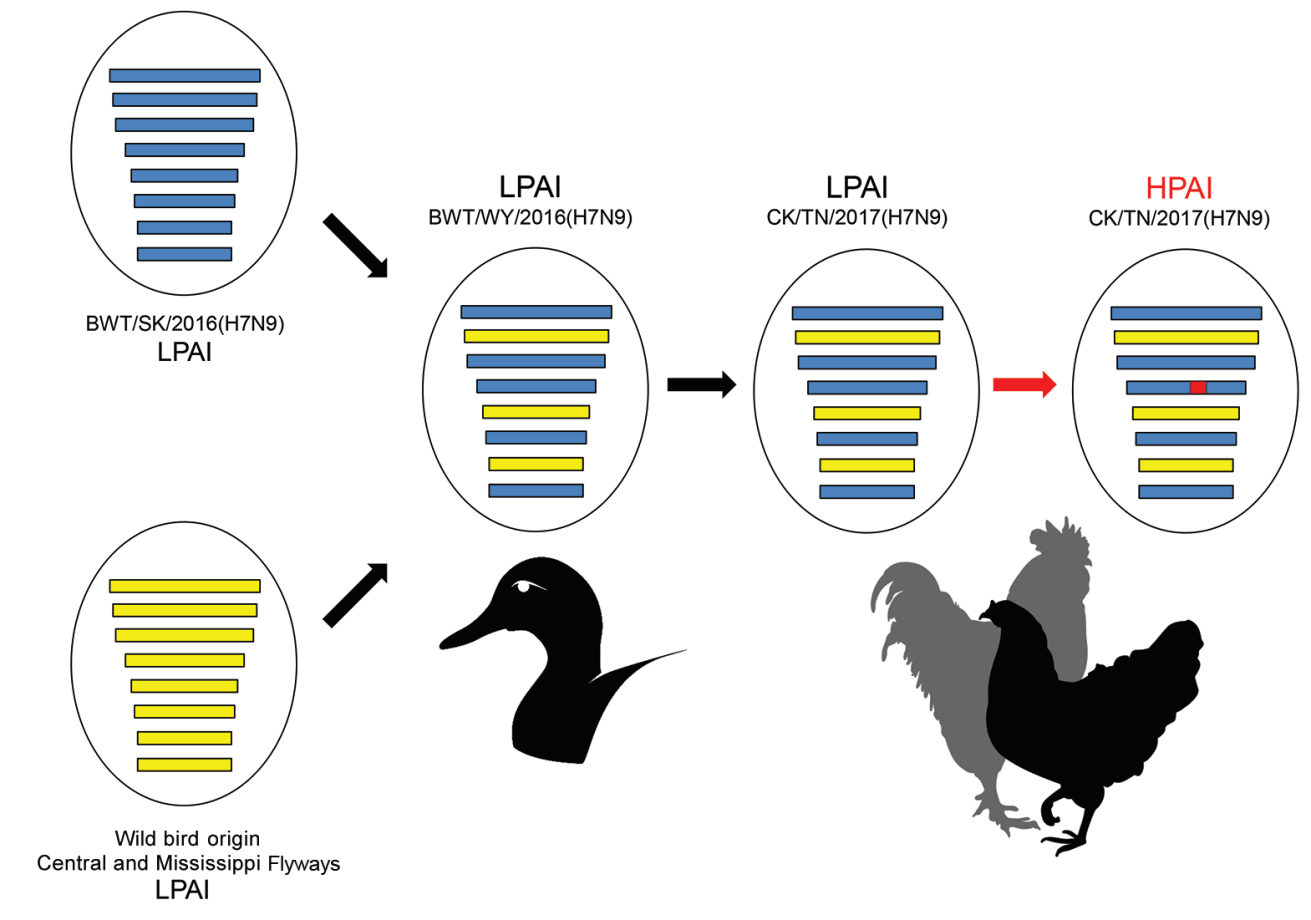
Genome constellation of influenza A(H7N9) viruses. Viruses are represented by ovals containing horizontal bars that represent the 8 influenza gene segments (from top to bottom: polymerase basic 2, polymerase basic 1, polymerase acidic, hemagglutinin, nucleoprotein, neuraminidase, matrix, and nonstructural). A genome reassortment event between the H7N9 virus from Saskatchewan, Canada (blue segments) and viruses from wild birds of the US Central and Mississippi Migratory Flyways (yellow segments) led to the genome assortment present in the Wyoming LPAI virus (BWT/WY/2016). The genome constellation of BWT/WY/2016 is the same as those of the Tennessee H7N9 viruses. A red bar in the hemagglutinin gene of the HPAI virus indicates the insertion at the hemagglutinin cleavage site. HPAI, highly pathogenic avian influenza; LPAI, low pathogenicity avian influenza.

An H7N9 LPAI closely related to the Tennessee and Alabama H7N9 viruses was previously detected on September 7, 2016, in a Wildlife Services wild bird surveillance pooled oropharyngeal and cloacal swab sample collected from a blue-winged teal in Goshen County, Wyoming, geographically located within the North American Central Migratory Flyway. The LPAI A/blue-winged teal/Wyoming/AH0099021/2016(H7N9) virus (BWT/WY/2016) shared a high level of nucleotide identity (>99.1%–99.8%) with the H7N9 outbreak strain across all 8 gene segments and appeared to be a precursor of the H7N9 HPAI virus that caused the outbreak among poultry (Figure 1; Technical Appendix Figures 3, 4). Surveillance in Canada led to the identification of a similar virus, A/blue-winged teal/Saskatchewan/109–701/2016(H7N9) (BWT/SK/2016), on August 12, 2016. In phylogenetic analyses, 5 genes (polymerase basic 2, polymerase acidic, HA, neuraminidase, nonstructural) from BWT/WY/2016, BWT/SK/2016, and the H7N9 outbreak isolates clustered together (Technical Appendix Figure 2, panels A–C, E, H). The polymerase basic 1, nucleoprotein, and matrix genes of BWT/WY/2016 and H7N9 outbreak isolates were derived from a variety of different North America lineage LPAI viruses circulating in the Central and Mississippi Migratory Flyways during 2016 (Technical Appendix Figure 2, panels D, F, G).

A previous report suggests that the genomes of North America wild bird lineage influenza A viruses are evolving through a remarkably high rate of genome reassortment, forming transient genome constellations that rapidly change with no apparent pattern of gene segment association (*6*). In contrast, a high level of nucleotide identity among all segments of the BWT/WY/2016 and H7N9 outbreak isolates (Technical Appendix Figure 4) suggests that this genome constellation might have been maintained in the wild bird population since its emergence in 2016, with subsequent dissemination to poultry in the southeastern states in 2017.

**Figure 2:**
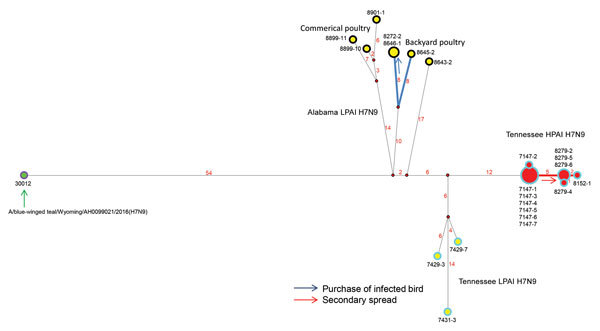
Median-joining phylogenetic network of influenza A(H7N9) viruses, United States, 2017. The median-joining network was constructed from concatenated H7N9 virus genomes containing all 8 segments. This network includes all the most parsimonious trees linking the sequences. Each unique sequence is represented by a circle sized relative to its frequency in the dataset. Isolates are colored according to the sample: red inner circle represents HPAI in poultry, yellow inner circle represents LPAI in poultry, green inner circle represents LPAI in a wild bird, purple outer circle represents isolates from Wyoming, black outer circle represents isolates from Alabama, and sky-blue outer circle represents isolates from Tennessee. Bold lines indicate farm-to-farm transmission verified by epidemiologic investigations. Red numbers indicate number of nucleotide changes between isolates. Black numbers are abbreviated isolate names. HPAI, highly pathogenic avian influenza; LPAI, low pathogenicity avian influenza.

The median-joining phylogenetic network analysis suggests separate introductions occurred from a common source into Tennessee and Alabama (Figure 2). The estimated time to most recent common ancestor of the HA genes was October 5, 2016 (95% Bayesian credible interval August 8, 2016–December 10, 2016) (Table), corresponding with the waterfowl fall migration season and time of BWT/WY/2016 sample collection. The LPAI viruses identified at Alabama broiler breeder farms were probably introduced separately. The network analysis also suggested that the HPAI and LPAI viruses identified at chicken farms in Tennessee probably originated from a common LPAI virus, and a single mutational event led to the HPAI virus that was detected at the first farm and subsequently spread to the second (Figure 2). An epidemiologic investigation revealed that delayed carcass disposal at the first site with HPAI virus might have facilitated transmission to the second and that the purchase of an infected guinea fowl caused transmission of an LPAI virus between 2 sites in Alabama. The 8-gene network analysis suggested the potential for >3 separate virus introductions: 1) LPAI in Alabama commercial poultry, 2) LPAI in backyard Alabama poultry, and 3) LPAI and HPAI in Tennessee commercial poultry. Bayesian phylogenetic analyses indicated high posterior probabilities (>0.98) for each of the clusters of viruses in these introductions; the time to most recent common ancestor of each of these 3 clusters was December 2016‒January 2017, corresponding to the wintering season for wild birds in this area (Table). The distribution of cases across 4 states and discovery of antibody-positive flocks with low or no viral RNA identified during routine and zone surveillance during March 2017 together with the phylogenetic analysis indicating a theoretical poultry precursor and variability in viruses from the same farm suggest that the H7N9 virus circulated in the area undetected in poultry before the initial HPAI virus detection.

**Table:** Times to most recent common ancestors of H7N9 viruses, Tennessee and Alabama, USA, March 2017*

Virus clusters (no. taxa)†	tMRCA‡	BCI 95%	Posterior probability
Tennessee and Alabama LPAI and HPAI viruses (22)	2016 Oct 5	2016 Aug 8–2016 Dec 10	1.00
Tennessee LPAI and HPAI viruses (15)	2016 Dec 21	2016 Nov 7–2017 Feb 3	1.00
Tennessee LPAI virus (3)	2017 Jan 23	2016 Dec 3–2017 Feb 25	0.98
Tennessee HPAI virus (12)	2017 Jan 30	2017 Jan 1–2017 Feb 25	1.00
Alabama LPAI virus from backyard guinea fowl (3)	2017 Jan 8	2016 Nov 18–2017 Feb 21	1.00
Alabama LPAI virus from commercial chicken (3)	2017 Jan 26	2016 Dec 10–2017 Mar 8	1.00

*BCI, Bayesian credible interval; HPAI, highly pathogenic avian influenza; LPAI, low pathogenicity avian influenza; tMRCA, time to most recent common ancestor.  †See online Technical Appendix Table (https://wwwnc.cdc.gov/EID/article/23/11/17-1013-Techapp1.pdf) for specific virus strains used in analysis. ‡Times to most recent common ancestors of hemagglutinin segment.

## Conclusions

Whole-genome sequencing and comparative genetic analyses of all available sequences of the North America wild bird H7N9 lineage suggest that the virus in the Wyoming blue-winged teal represents a precursor to the poultry viruses in the southeastern United States and that the mutation from an LPAI virus to an HPAI virus occurred in poultry. Our data suggest that the virus circulated in the region undetected in poultry before the initial HPAI virus detection and that >3 separate virus introductions occurred. These findings highlight the need for routine and frequent testing of poultry for avian influenza virus because reportable LPAI viruses might circulate without causing any clinical signs.

Technical Appendix Description of methods, list and location of isolates, and phylogenetic analyses (maximum-likelihood, relaxed clock, and sequence alignment) of all 8 genome segments of the highly pathogenic and low pathogenicity avian influenza viruses identified in Tennessee and Alabama, United States, March 2017.
