# Supplementary material for: Highly Pathogenic Avian Influenza A(H7N9) Virus, Tennessee, USA, March 2017
Source: Emerg Infect Dis. 2017 Nov;23(11):1860–3. doi: 10.3201/eid2311.171013 (PMC5652434; doi:10.3201/eid2311.171013)
Supplement: Technical Appendix — Description of methods, list and location of isolates, and phylogenetic analyses (maximum-likelihood, relaxed clock, and sequence alignment) of all 8 genome segments of the highly pathogenic and low pathogenicity avian influenza viruses identified in Tennessee and Alabama, United States, March 2017. [file 17-1013-Techapp-s1.pdf]

Article DOI: https://doi.org/10.3201/eid2311.171013

Highly Pathogenic Avian Influenza A(H7N9) Virus, Tennessee, USA, March 2017

Technical Appendix

Methods for Genome Sequencing and Phylogenetic Analysis

Nucleotide Sequencing

Complete genomes of 12 highly pathogenic and 10 low pathogenicity avian influenza viruses were sequenced in this study. We extracted viral RNA from samples using the MagMAX Viral RNA Isolation Kit (Ambion, Austin, TX, USA) and synthesized complementary DNA by performing reverse transcription with SuperScript III (Invitrogen, Carlsbad, CA, USA). We amplified all 8 segments of the isolates by PCR and conducted complete genome sequencing using the Miseq system (Illumina, San Diego, CA, USA) and Ion Torrent Personal Genome Machine (PGM) (Thermo Fisher Scientific, Waltham, MA, USA). For the Illumina Miseq system, the Nextera XT DNA Sample Preparation Kit (Illumina) was used according to the manufacturer's instructions to generate multiplexed paired-end sequencing libraries. The dsDNA was fragmented and tagged with adapters by Nextera XT transposase and 12-cycle PCR amplification. We purified fragments on Agencourt AMpure XP beads (Beckman Coulter, Fullerton, CA, USA) and analyzed them with a High Sensitivity DNA Chip and Bioanalyzer (Agilent Technologies, Santa Clara, CA, USA). The barcoded multiplexed library sequencing was performed by using the 300 cycle MiSeq Reagent Kit v2 (Illumina). For Ion Torrent PGM, we purified the PCR product and prepared the DNA libraries with the IonXpress Plus Fragment Library Kit with Ion Xpress barcode adapters. Prepared libraries were quantitated using the Bioanalyzer DNA 1000 (Agilent Technologies). We diluted the quantitated libraries and pooled them for library amplification using the Ion One Touch 2 System and Enrichment System. Following enrichment, DNA was loaded onto an Ion 314 or Ion 316 chip and sequenced using the Ion PGM 200 v2 Sequencing Kit. De novo and directed assembly of genome sequences were carried out using the SeqMan NGen v4 program (http://www.dnastar.com/t-nextgen-seqman-

ngen.aspx). Nucleotide sequences were deposited in GenBank (accession nos. KY818808-KY818839 and MF357713-MF357856).

Phylogenetic Analysis

We generated maximum-likelihood phylogenies of each gene segment using RAxML and the general time-reversible nucleotide substitution model, with among-site rate variation modeled by using a discrete gamma distribution (*1*). We generated bootstrap support values using 1,000 rapid bootstrap replicates. We performed Bayesian relaxed clock phylogenetic analysis of the hemagglutinin (HA) gene using BEAST v1.8.3 (*2*). We applied an uncorrelated log-normal distribution relaxed clock method, the Hasegawa–Kishino–Yano nucleotide substitution model, and the Bayesian skyline coalescent prior. A Markov Chain Monte Carlo method to sample trees and evolutionary parameters was run for 50 million generations. At least 3 independent chains were combined to ensure adequate sampling of the posterior distribution of trees. BEAST output was analyzed with TRACER v1.4 (https://beast.bio.ed.ac.uk/tracer) with 5% burn-in. A maximum clade credibility tree was generated for each data set by using TreeAnnotator in BEAST. We used FigTree v1.4.2 (https://tree.bio.ed.ac.uk/) to visualize trees. To better visualize the genetic relatedness of viruses, all available full genome sequences were concatenated, with the highly pathogenic avian influenza virus modified to remove the nucleotide insertion at the HA cleavage site, and analyzed using the median-joining method implemented by NETWORK v5.0 (*3*).

Serology

Any antibody detection by National Poultry Improvement Program–authorized laboratories using influenza A serologic testing (ELISA or agar gel immunodiffusion test) was forwarded to the National Veterinary Services Laboratories (Ames, Iowa, USA) for confirmation testing by HA (H1-H16) and neuraminidase (N1-N9) inhibition testing.

References

1. Stamatakis A. RAxML version 8: a tool for phylogenetic analysis and post-analysis of large phylogenies. Bioinformatics. 2014;30:1312–3. PubMed http://dx.doi.org/10.1093/bioinformatics/btu033

2. Drummond AJ, Rambaut A. BEAST: Bayesian evolutionary analysis by sampling trees. BMC Evol Biol. 2007;7:214. PubMed http://dx.doi.org/10.1186/1471-2148-7-214

3. Bandelt HJ, Forster P, Röhl A. Median-joining networks for inferring intraspecific phylogenies. Mol Biol Evol. 1999;16:37–48. PubMed http://dx.doi.org/10.1093/oxfordjournals.molbev.a026036

Technical Appendix Table. Avian influenza viruses used in study, Tennessee and Alabama, United States, March 2017

Site	Date collected	Virus strain designation	County	Pathotype
A	2017 Mar 3	A/chicken/Tennessee/17–007147–1/2017(H7N9), A/chicken/Tennessee/17–007147–2/2017(H7N9), A/chicken/Tennessee/17–007147–3/2017(H7N9), A/chicken/Tennessee/17–007147–4/2017(H7N9), A/chicken/Tennessee/17–007147–5/2017(H7N9), A/chicken/Tennessee/17–007147–6/2017(H7N9), A/chicken/Tennessee/17–007147–7/2017(H7N9)	Lincoln	HPAI
B	2017 Mar 13	A/chicken/Tennessee/17–008152–1/2017(H7N9)	Lincoln	HPAI
B	2017 Mar 14	A/chicken/Tennessee/17–008279–2/2017(H7N9), A/chicken/Tennessee/17–008279–4/2017(H7N9), A/chicken/Tennessee/17–008279–5/2017(H7N9), A/chicken/Tennessee/17–008279–6/2017(H7N9)	Lincoln	HPAI
C	2017 Mar 6	A/chicken/Tennessee/17–007429–3/2017(H7N9), A/chicken/Tennessee/17–007429–7/2017(H7N9)	Giles	LPAI
C	2017 Mar 6	A/chicken/Tennessee/17–007431–3/2017(H7N9)	Giles	LPAI
D	2017 Mar 12	A/guinea_fowl/Alabama/17–008272–2/2017(H7N9)	Jackson	LPAI
E	2017 Mar 15	A/duck/Alabama/17–008643–2/2017(H7N9)	Madison	LPAI
F	2017 Mar 14	A/guinea_fowl/Alabama/17–008645–2/2017(H7N9)	Jackson	LPAI
G	2017 Mar 14	A/guinea_fowl/Alabama/17–008646–1/2017(H7N9)	DeKalb	LPAI
H	2017 Mar 19	A/chicken/Alabama/17–008899–10/2017(H7N9), A/chicken/Alabama/17–008899–11/2017(H7N9)	Cullman	LPAI
H	2017 Mar 17	A/chicken/Alabama/17–008901–1/2017(H7N9)	Cullman	LPAI

*HPAI, highly pathogenic avian influenza; LPAI, low pathogenicity avian influenza. Six additional premises in Alabama, Tennessee, Kentucky, and Georgia were identified as H7N9 LPAI affected, but insufficient viral RNA prevented virus sequencing and analysis.

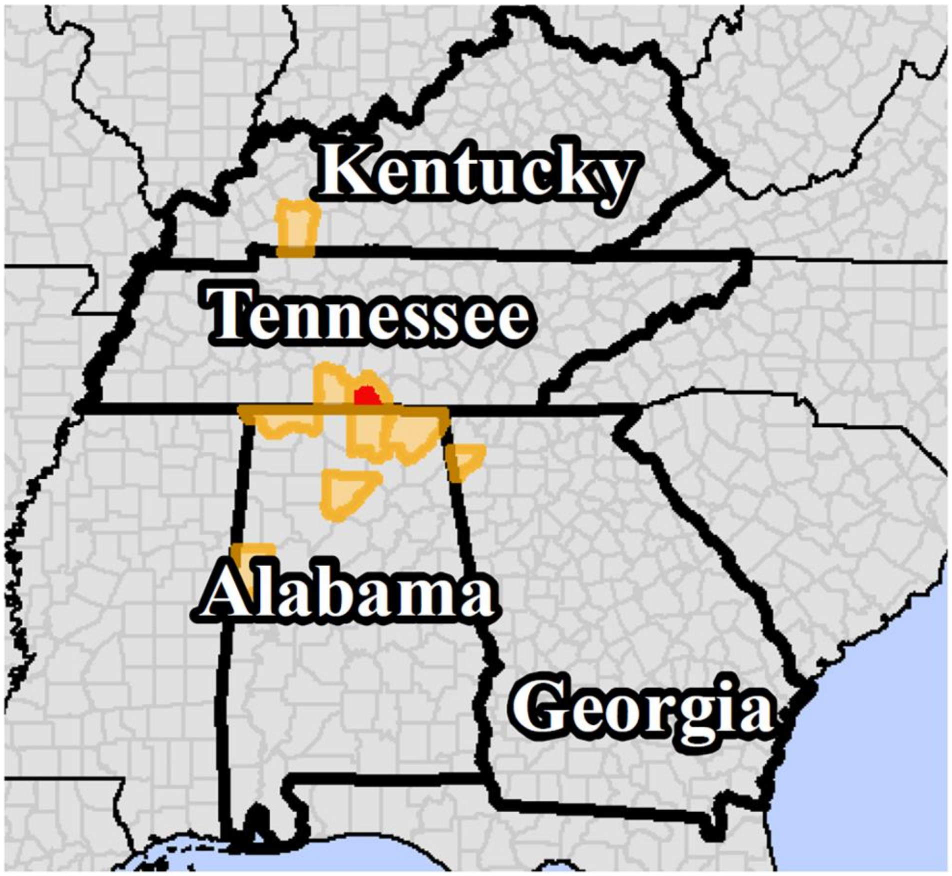

Technical Appendix Figure 1. Outbreak map of H7N9 low pathogenicity (yellow) and highly pathogenic (red) avian influenza.

Technical Appendix Figure 2 (following pages). Maximum-likelihood phylogeny of H7N9 virus genes A) hemagglutinin, B) neuraminidase, C) polymerase basic 2, D) polymerase basic 1, E) polymerase acidic, F) nucleoprotein, G) matrix, and H) nonstructural. Numbers along branches indicate bootstrap values >70%. Brackets indicate the genetic subgroups. Scale bar indicates nucleotide substitutions per site. The following 4 symbols were used to indicate avian influenza H7N9 viruses: black circle for highly pathogenic H7N9, open circle for low pathogenicity H7N9, black triangle for A/blue-winged teal/Wyoming/AH0099021/2016, and open triangle for A/blue-winged teal/Saskatchewan/109–701/2016. HA, hemagglutinin; M, matrix; NA, neuraminidase; NP, nucleoprotein; NS, nonstructural; PA, polymerase acidic; PB1, polymerase basic 1; PB2, polymerase basic 2.

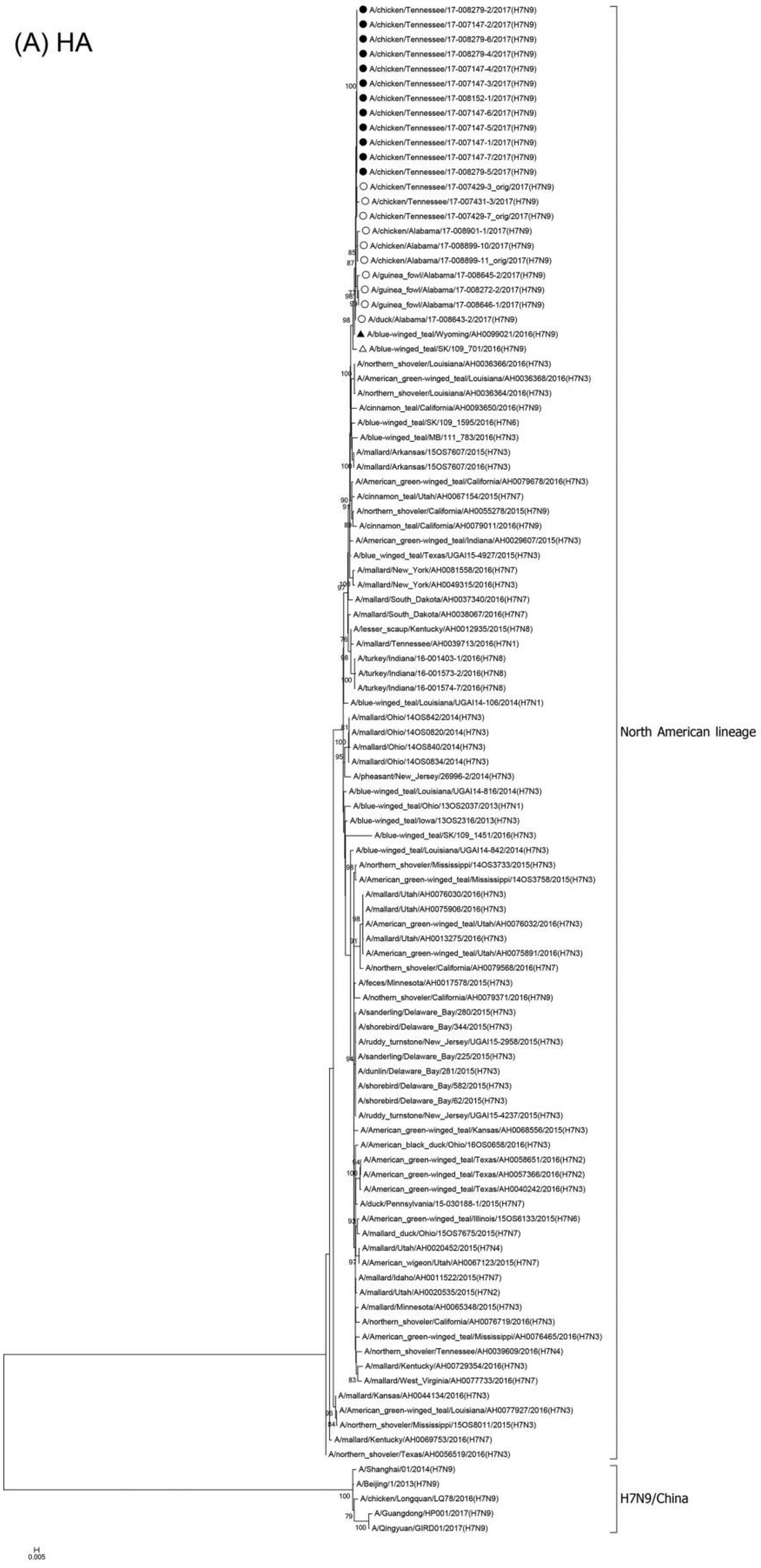

(A) HA
A/chicken/Tennessee/17-008279-2/2017(H7N9)
A/chicken/Tennessee/17-007147-2/2017(H7N9)
A/chicken/Tennessee/17-008279-6/2017(H7N9)
A/chicken/Tennessee/17-008279-4/2017(H7N9)
A/chicken/Tennessee/17-007147-4/2017(H7N9)
A/chicken/Tennessee/17-007147-3/2017(H7N9)
A/chicken/Tennessee/17-008152-1/2017(H7N9)
A/chicken/Tennessee/17-007147-6/2017(H7N9)
A/chicken/Tennessee/17-007147-5/2017(H7N9)
A/chicken/Tennessee/17-007147-1/2017(H7N9)
A/chicken/Tennessee/17-007147-7/2017(H7N9)
A/chicken/Tennessee/17-008279-5/2017(H7N9)
A/chicken/Tennessee/17-007429-3_orig/2017(H7N9)
A/chicken/Tennessee/17-007431-3/2017(H7N9)
A/chicken/Tennessee/17-007429-7_orig/2017(H7N9)
A/chicken/Alabama/17-008901-1/2017(H7N9)
A/chicken/Alabama/17-008899-10/2017(H7N9)
A/chicken/Alabama/17-008899-11_orig/2017(H7N9)
A/guinea_fowl/Alabama/17-008645-2/2017(H7N9)
A/guinea_fowl/Alabama/17-008272-2/2017(H7N9)
A/guinea_fowl/Alabama/17-008646-1/2017(H7N9)
A/duck/Alabama/17-008643-2/2017(H7N9)
A/blue-winged_teal/Wyoming/AH0099021/2016(H7N9)
A/blue-winged_teal/SK/109_701/2016(H7N9)
A/northern_shoveler/Louisiana/AH0036366/2016(H7N3)
A/American_green-winged_teal/Louisiana/AH0036368/2016(H7N3)
A/northern_shoveler/Louisiana/AH0036364/2016(H7N3)
A/cinnamon_teal/California/AH0093650/2016(H7N9)
A/blue-winged_teal/SK/109_1595/2016(H7N6)
A/blue-winged_teal/MB/111_783/2016(H7N3)
A/mallard/Arkansas/15OS7607/2015(H7N3)
A/mallard/Arkansas/15OS7607/2016(H7N3)
A/American_green-winged_teal/California/AH0079678/2016(H7N3)
A/cinnamon_teal/Utah/AH0067154/2015(H7N7)
A/northern_shoveler/California/AH0055278/2015(H7N9)
A/cinnamon_teal/California/AH0079011/2016(H7N9)
A/American_green-winged_teal/Indiana/AH0029607/2015(H7N3)
A/blue_winged_teal/Texas/UGAI15-4927/2015(H7N3)
A/mallard/New_York/AH0081558/2016(H7N7)
A/mallard/New_York/AH0049315/2016(H7N3)
A/mallard/South_Dakota/AH0037340/2016(H7N7)
A/mallard/South_Dakota/AH0038067/2016(H7N7)
A/lesser_scaup/Kentucky/AH0012935/2015(H7N8)
A/mallard/Tennessee/AH0039713/2016(H7N1)
A/turkey/Indiana/16-001403-1/2016(H7N8)
A/turkey/Indiana/16-001573-2/2016(H7N8)
A/turkey/Indiana/16-001574-7/2016(H7N8)
A/blue-winged_teal/Louisiana/UGAI14-106/2014(H7N1)
A/mallard/Ohio/14OS842/2014(H7N3)
A/mallard/Ohio/14OS0820/2014(H7N3)
A/mallard/Ohio/14OS840/2014(H7N3)
A/mallard/Ohio/14OS0834/2014(H7N3)
A/pheasant/New_Jersey/26996-2/2014(H7N3)
A/blue-winged_teal/Louisiana/UGAI14-816/2014(H7N3)
A/blue-winged_teal/Ohio/13OS2037/2013(H7N1)
A/blue-winged_teal/Iowa/13OS2316/2013(H7N3)
A/blue-winged_teal/SK/109_1451/2016(H7N3)
A/blue-winged_teal/Louisiana/UGAI14-842/2014(H7N3)
A/northern_shoveler/Mississippi/14OS3733/2015(H7N3)
A/American_green-winged_teal/Mississippi/14OS3758/2015(H7N3)
A/mallard/Utah/AH0076030/2016(H7N3)
A/mallard/Utah/AH0075906/2016(H7N3)
A/American_green-winged_teal/Utah/AH0076032/2016(H7N3)
A/mallard/Utah/AH0013275/2016(H7N3)
A/American_green-winged_teal/Utah/AH0075891/2016(H7N3)
A/northern_shoveler/California/AH0079568/2016(H7N7)
A/feces/Minnesota/AH0017578/2015(H7N3)
A/nothern_shoveler/California/AH0079371/2016(H7N9)
A/sanderling/Delaware_Bay/280/2015(H7N3)
A/shorebird/Delaware_Bay/344/2015(H7N3)
A/ruddy_turnstone/New_Jersey/UGAI15-2958/2015(H7N3)
A/sanderling/Delaware_Bay/225/2015(H7N3)
A/dunlin/Delaware_Bay/281/2015(H7N3)
A/shorebird/Delaware_Bay/582/2015(H7N3)
A/shorebird/Delaware_Bay/62/2015(H7N3)
A/ruddy_turnstone/New_Jersey/UGAI15-4237/2015(H7N3)
A/American_green-winged_teal/Kansas/AH0068556/2015(H7N3)
A/American_black_duck/Ohio/16OS0658/2016(H7N3)
A/American_green-winged_teal/Texas/AH0058651/2016(H7N2)
A/American_green-winged_teal/Texas/AH0057366/2016(H7N2)
A/American_green-winged_teal/Texas/AH0040242/2016(H7N3)
A/duck/Pennsylvania/15-030188-1/2015(H7N7)
A/American_green-winged_teal/Illinois/15OS6133/2015(H7N6)
A/mallard_duck/Ohio/15OS7675/2015(H7N7)
A/mallard/Utah/AH0020452/2015(H7N4)
A/American_wigeon/Utah/AH0067123/2015(H7N7)
A/mallard/Idaho/AH0011522/2015(H7N7)
A/mallard/Utah/AH0020535/2015(H7N2)
A/mallard/Minnesota/AH0065348/2015(H7N3)
A/northern_shoveler/California/AH0076719/2016(H7N3)
A/American_green-winged_teal/Mississippi/AH0076465/2016(H7N3)
A/northern_shoveler/Tennessee/AH0039609/2016(H7N4)
A/mallard/Kentucky/AH00729354/2016(H7N3)
A/mallard/West_Virginia/AH0077733/2016(H7N7)
A/mallard/Kansas/AH0044134/2016(H7N3)
A/American_green-winged_teal/Louisiana/AH0077927/2016(H7N3)
A/northern_shoveler/Mississippi/15OS8011/2015(H7N3)
A/mallard/Kentucky/AH0069753/2016(H7N7)
A/northern_shoveler/Texas/AH0056519/2016(H7N3)
North American lineage
A/Shanghai/01/2014(H7N9)
A/Beijing/1/2013(H7N9)
A/chicken/Longquan/LQ78/2016(H7N9)
A/Guangdong/HP001/2017(H7N9)
A/Qingyuan/GIRD01/2017(H7N9)
H7N9/China
0.005

(B) NA

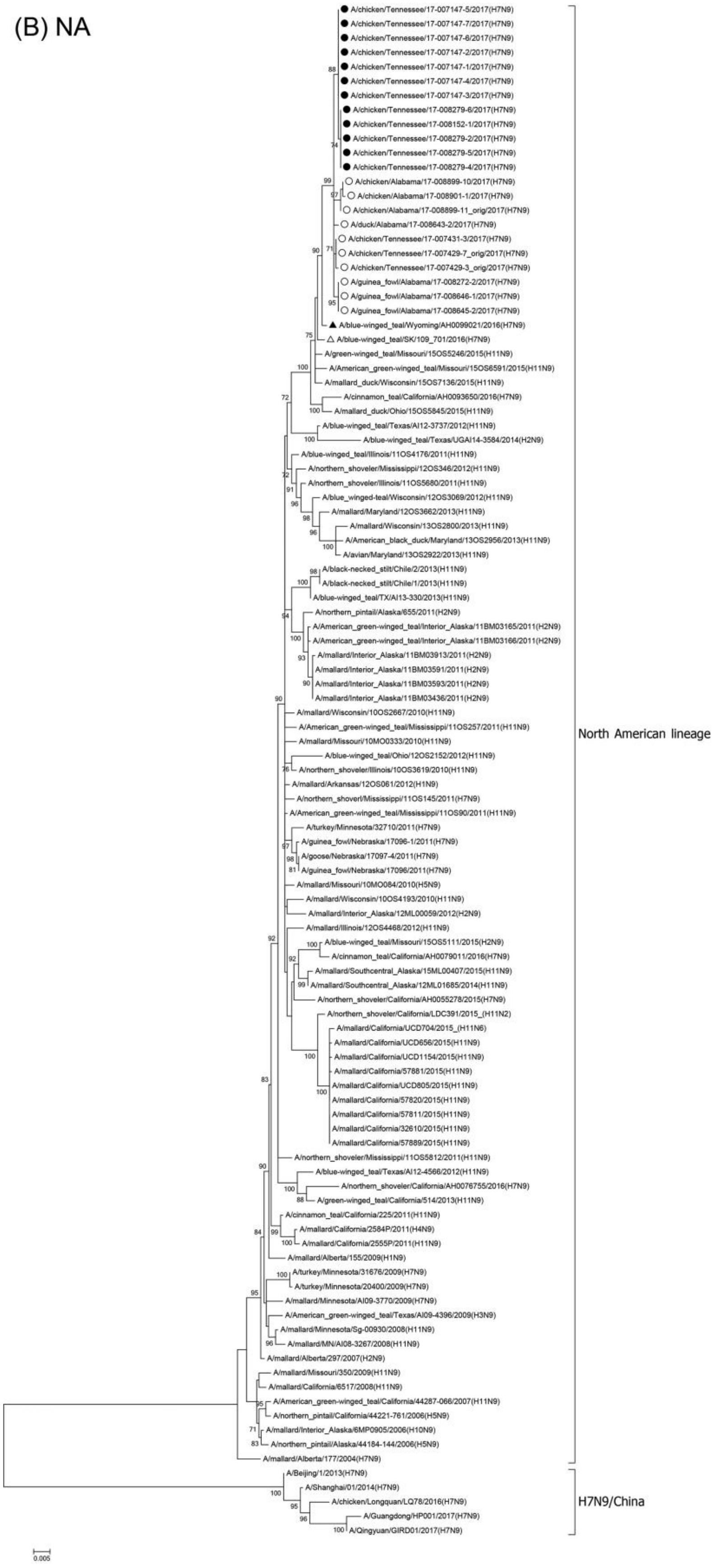

A/chicken/Tennessee/17-007147-5/2017(H7N9)
A/chicken/Tennessee/17-007147-7/2017(H7N9)
A/chicken/Tennessee/17-007147-6/2017(H7N9)
A/chicken/Tennessee/17-007147-2/2017(H7N9)
A/chicken/Tennessee/17-007147-1/2017(H7N9)
A/chicken/Tennessee/17-007147-4/2017(H7N9)
A/chicken/Tennessee/17-007147-3/2017(H7N9)
A/chicken/Tennessee/17-008279-6/2017(H7N9)
A/chicken/Tennessee/17-008152-1/2017(H7N9)
A/chicken/Tennessee/17-008279-2/2017(H7N9)
A/chicken/Tennessee/17-008279-5/2017(H7N9)
A/chicken/Tennessee/17-008279-4/2017(H7N9)
A/chicken/Alabama/17-008899-10/2017(H7N9)
A/chicken/Alabama/17-008901-1/2017(H7N9)
A/chicken/Alabama/17-008899-11_orig/2017(H7N9)
A/duck/Alabama/17-008643-2/2017(H7N9)
A/chicken/Tennessee/17-007431-3/2017(H7N9)
A/chicken/Tennessee/17-007429-7_orig/2017(H7N9)
A/chicken/Tennessee/17-007429-3_orig/2017(H7N9)
A/guinea_fowl/Alabama/17-008272-2/2017(H7N9)
A/guinea_fowl/Alabama/17-008646-1/2017(H7N9)
A/guinea_fowl/Alabama/17-008645-2/2017(H7N9)
A/blue-winged_teal/Wyoming/AH0099021/2016(H7N9)
A/blue-winged_teal/SK/109_701/2016(H7N9)
A/green-winged_teal/Missouri/15OS5246/2015(H11N9)
A/American_green-winged_teal/Missouri/15OS6591/2015(H11N9)
A/mallard_duck/Wisconsin/15OS7136/2015(H11N9)
A/cinnamon_teal/California/AH0093650/2016(H7N9)
A/mallard_duck/Ohio/15OS5845/2015(H11N9)
A/blue-winged_teal/Texas/AI12-3737/2012(H11N9)
A/blue-winged_teal/Texas/UGAI14-3584/2014(H2N9)
A/blue-winged_teal/Illinois/11OS4176/2011(H11N9)
A/northern_shoveler/Mississippi/12OS346/2012(H11N9)
A/northern_shoveler/Illinois/11OS5680/2011(H11N9)
A/blue_winged-teal/Wisconsin/12OS3069/2012(H11N9)
A/mallard/Maryland/12OS3662/2013(H11N9)
A/mallard/Wisconsin/13OS2800/2013(H11N9)
A/American_black_duck/Maryland/13OS2956/2013(H11N9)
A/avian/Maryland/13OS2922/2013(H11N9)
A/black-necked_stilt/Chile/2/2013(H11N9)
A/black-necked_stilt/Chile/1/2013(H11N9)
A/blue-winged_teal/TX/AI13-330/2013(H11N9)
A/northern_pintail/Alaska/655/2011(H2N9)
A/American_green-winged_teal/Interior_Alaska/11BM03165/2011(H2N9)
A/American_green-winged_teal/Interior_Alaska/11BM03166/2011(H2N9)
A/mallard/Interior_Alaska/11BM03913/2011(H2N9)
A/mallard/Interior_Alaska/11BM03591/2011(H2N9)
A/mallard/Interior_Alaska/11BM03593/2011(H2N9)
A/mallard/Interior_Alaska/11BM03436/2011(H2N9)
A/mallard/Wisconsin/10OS2667/2010(H11N9)
A/American_green-winged_teal/Mississippi/11OS257/2011(H11N9)
A/mallard/Missouri/10MO0333/2010(H11N9)
A/blue-winged_teal/Ohio/12OS2152/2012(H11N9)
A/northern_shoveler/Illinois/10OS3619/2010(H11N9)
A/mallard/Arkansas/12OS061/2012(H1N9)
A/northern_shoverl/Mississippi/11OS145/2011(H7N9)
A/American_green-winged_teal/Mississippi/11OS90/2011(H11N9)
A/turkey/Minnesota/32710/2011(H7N9)
A/guinea_fowl/Nebraska/17096-1/2011(H7N9)
A/goose/Nebraska/17097-4/2011(H7N9)
A/guinea_fowl/Nebraska/17096/2011(H7N9)
A/mallard/Missouri/10MO084/2010(H5N9)
A/mallard/Wisconsin/10OS4193/2010(H11N9)
A/mallard/Interior_Alaska/12ML00059/2012(H2N9)
A/mallard/Illinois/12OS4468/2012(H11N9)
A/blue-winged_teal/Missouri/15OS5111/2015(H2N9)
A/cinnamon_teal/California/AH0079011/2016(H7N9)
A/mallard/Southcentral_Alaska/15ML00407/2015(H11N9)
A/mallard/Southcentral_Alaska/12ML01685/2014(H11N9)
A/northern_shoveler/California/AH0055278/2015(H7N9)
A/northern_shoveler/California/LDC391/2015_(H11N2)
A/mallard/California/UCD704/2015_(H11N6)
A/mallard/California/UCD656/2015(H11N9)
A/mallard/California/UCD1154/2015(H11N9)
A/mallard/California/57881/2015(H11N9)
A/mallard/California/UCD805/2015(H11N9)
A/mallard/California/57820/2015(H11N9)
A/mallard/California/57811/2015(H11N9)
A/mallard/California/32610/2015(H11N9)
A/mallard/California/57889/2015(H11N9)
A/northern_shoveler/Mississippi/11OS5812/2011(H11N9)
A/blue-winged_teal/Texas/AI12-4566/2012(H11N9)
A/northern_shoveler/California/AH0076755/2016(H7N9)
A/green-winged_teal/California/514/2013(H11N9)
A/cinnamon_teal/California/225/2011(H11N9)
A/mallard/California/2584P/2011(H4N9)
A/mallard/California/2555P/2011(H11N9)
A/mallard/Alberta/155/2009(H1N9)
A/turkey/Minnesota/31676/2009(H7N9)
A/turkey/Minnesota/20400/2009(H7N9)
A/mallard/Minnesota/AI09-3770/2009(H7N9)
A/American_green-winged_teal/Texas/AI09-4396/2009(H3N9)
A/mallard/Minnesota/Sg-00930/2008(H11N9)
A/mallard/MN/AI08-3267/2008(H11N9)
A/mallard/Alberta/297/2007(H2N9)
A/mallard/Missouri/350/2009(H11N9)
A/mallard/California/6517/2008(H11N9)
A/American_green-winged_teal/California/44287-066/2007(H11N9)
A/northern_pintail/California/44221-761/2006(H5N9)
A/mallard/Interior_Alaska/6MP0905/2006(H10N9)
A/northern_pintail/Alaska/44184-144/2006(H5N9)
A/mallard/Alberta/177/2004(H7N9)
A/Beijing/1/2013(H7N9)
A/Shanghai/01/2014(H7N9)
A/chicken/Longquan/LQ78/2016(H7N9)
A/Guangdong/HP001/2017(H7N9)
A/Qingyuan/GIRD01/2017(H7N9)
North American lineage
H7N9/China
0.005

(C) PB2

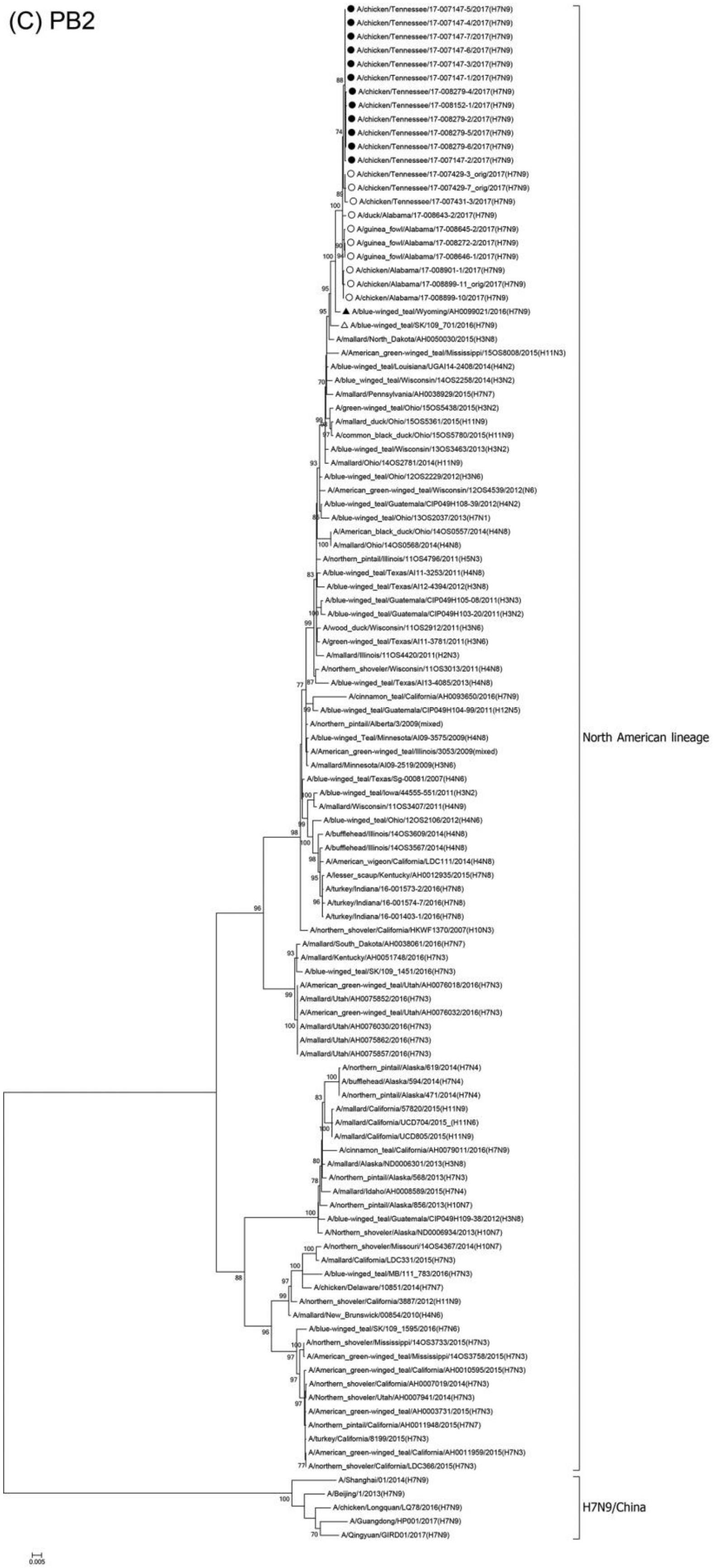

(D) PB1

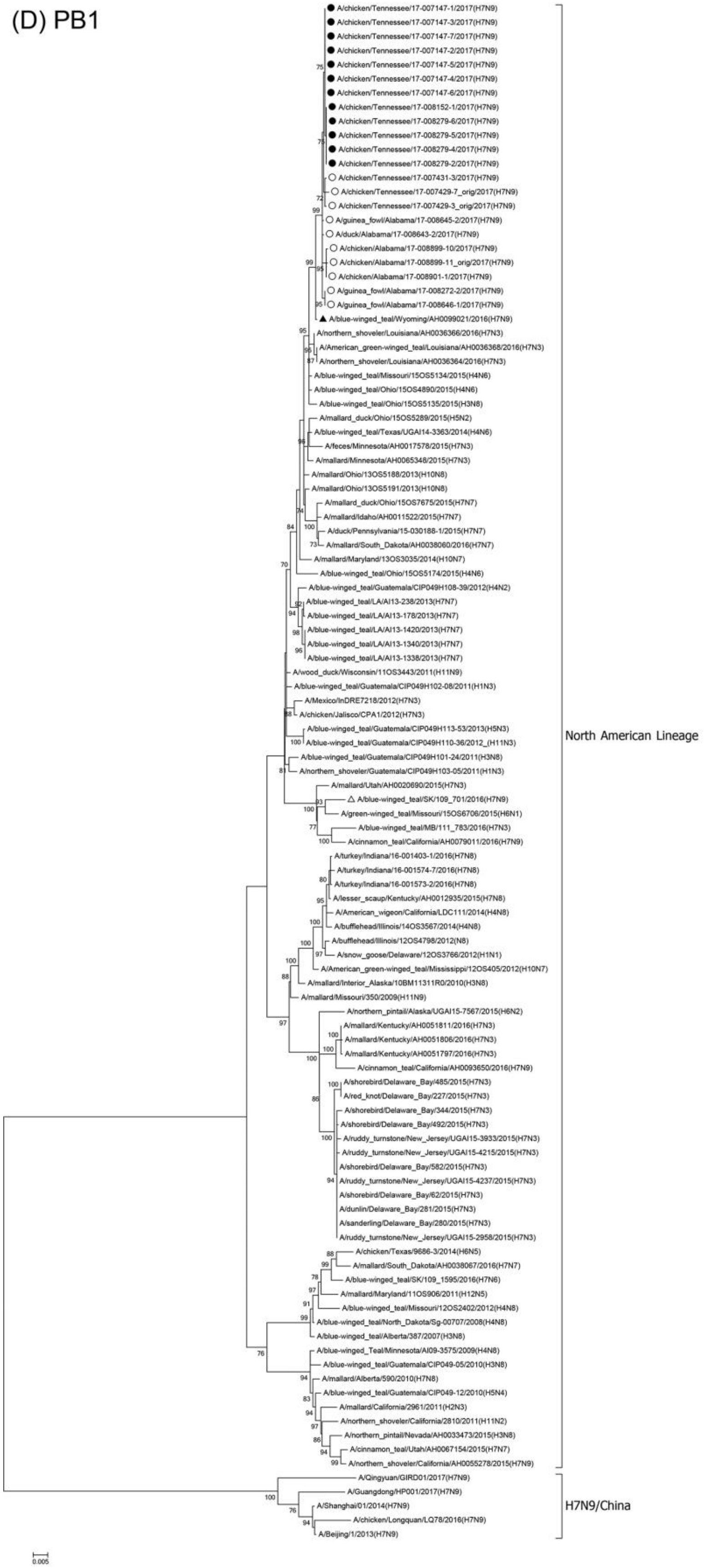
A/chicken/Tennessee/17-007147-1/2017(H7N9)
A/chicken/Tennessee/17-007147-3/2017(H7N9)
A/chicken/Tennessee/17-007147-7/2017(H7N9)
A/chicken/Tennessee/17-007147-2/2017(H7N9)
A/chicken/Tennessee/17-007147-5/2017(H7N9)
A/chicken/Tennessee/17-007147-4/2017(H7N9)
A/chicken/Tennessee/17-007147-6/2017(H7N9)
A/chicken/Tennessee/17-008152-1/2017(H7N9)
A/chicken/Tennessee/17-008279-6/2017(H7N9)
A/chicken/Tennessee/17-008279-5/2017(H7N9)
A/chicken/Tennessee/17-008279-4/2017(H7N9)
A/chicken/Tennessee/17-008279-2/2017(H7N9)
A/chicken/Tennessee/17-007431-3/2017(H7N9)
A/chicken/Tennessee/17-007429-7_orig/2017(H7N9)
A/chicken/Tennessee/17-007429-3_orig/2017(H7N9)
A/guinea_fowl/Alabama/17-008645-2/2017(H7N9)
A/duck/Alabama/17-008643-2/2017(H7N9)
A/chicken/Alabama/17-008899-10/2017(H7N9)
A/chicken/Alabama/17-008899-11_orig/2017(H7N9)
A/chicken/Alabama/17-008901-1/2017(H7N9)
A/guinea_fowl/Alabama/17-008272-2/2017(H7N9)
A/guinea_fowl/Alabama/17-008646-1/2017(H7N9)
A/blue-winged_teal/Wyoming/AH0099021/2016(H7N9)
A/northern_shoveler/Louisiana/AH0036366/2016(H7N3)
A/American_green-winged_teal/Louisiana/AH0036368/2016(H7N3)
A/northern_shoveler/Louisiana/AH0036364/2016(H7N3)
A/blue-winged_teal/Missouri/15OS5134/2015(H4N6)
A/blue-winged_teal/Ohio/15OS4890/2015(H4N6)
A/blue-winged_teal/Ohio/15OS5135/2015(H3N8)
A/mallard_duck/Ohio/15OS5289/2015(H5N2)
A/blue-winged_teal/Texas/UGAI14-3363/2014(H4N6)
A/feces/Minnesota/AH0017578/2015(H7N3)
A/mallard/Minnesota/AH0065348/2015(H7N3)
A/mallard/Ohio/13OS5188/2013(H10N8)
A/mallard/Ohio/13OS5191/2013(H10N8)
A/mallard_duck/Ohio/15OS7675/2015(H7N7)
A/mallard/Idaho/AH0011522/2015(H7N7)
A/duck/Pennsylvania/15-030188-1/2015(H7N7)
A/mallard/South_Dakota/AH0038060/2016(H7N7)
A/mallard/Maryland/13OS3035/2014(H10N7)
A/blue-winged_teal/Ohio/15OS5174/2015(H4N6)
A/blue-winged_teal/Guatemala/CIP049H108-39/2012(H4N2)
A/blue-winged_teal/LA/AI13-238/2013(H7N7)
A/blue-winged_teal/LA/AI13-178/2013(H7N7)
A/blue-winged_teal/LA/AI13-1420/2013(H7N7)
A/blue-winged_teal/LA/AI13-1340/2013(H7N7)
A/blue-winged_teal/LA/AI13-1338/2013(H7N7)
A/wood_duck/Wisconsin/11OS3443/2011(H11N9)
A/blue-winged_teal/Guatemala/CIP049H102-08/2011(H1N3)
A/Mexico/InDRE7218/2012(H7N3)
A/chicken/Jalisco/CPA1/2012(H7N3)
A/blue-winged_teal/Guatemala/CIP049H113-53/2013(H5N3)
A/blue-winged_teal/Guatemala/CIP049H110-36/2012_(H11N3)
A/blue-winged_teal/Guatemala/CIP049H101-24/2011(H3N8)
A/northern_shoveler/Guatemala/CIP049H103-05/2011(H1N3)
A/mallard/Utah/AH0020690/2015(H7N3)
A/blue-winged_teal/SK/109_701/2016(H7N9)
A/green-winged_teal/Missouri/15OS6706/2015(H6N1)
A/blue-winged_teal/MB/111_783/2016(H7N3)
A/cinnamon_teal/California/AH0079011/2016(H7N9)
A/turkey/Indiana/16-001403-1/2016(H7N8)
A/turkey/Indiana/16-001574-7/2016(H7N8)
A/turkey/Indiana/16-001573-2/2016(H7N8)
A/lesser_scaup/Kentucky/AH0012935/2015(H7N8)
A/American_wigeon/California/LDC111/2014(H4N8)
A/bufflehead/Illinois/14OS3567/2014(H4N8)
A/bufflehead/Illinois/12OS4798/2012(N8)
A/snow_goose/Delaware/12OS3766/2012(H1N1)
A/American_green-winged_teal/Mississippi/12OS405/2012(H10N7)
A/mallard/Interior_Alaska/10BM11311R0/2010(H3N8)
A/mallard/Missouri/350/2009(H11N9)
A/northern_pintail/Alaska/UGAI15-7567/2015(H6N2)
A/mallard/Kentucky/AH0051811/2016(H7N3)
A/mallard/Kentucky/AH0051806/2016(H7N3)
A/mallard/Kentucky/AH0051797/2016(H7N3)
A/cinnamon_teal/California/AH0093650/2016(H7N9)
A/shorebird/Delaware_Bay/485/2015(H7N3)
A/red_knot/Delaware_Bay/227/2015(H7N3)
A/shorebird/Delaware_Bay/344/2015(H7N3)
A/shorebird/Delaware_Bay/492/2015(H7N3)
A/ruddy_turnstone/New_Jersey/UGAI15-3933/2015(H7N3)
A/ruddy_turnstone/New_Jersey/UGAI15-4215/2015(H7N3)
A/shorebird/Delaware_Bay/582/2015(H7N3)
A/ruddy_turnstone/New_Jersey/UGAI15-4237/2015(H7N3)
A/shorebird/Delaware_Bay/62/2015(H7N3)
A/dunlin/Delaware_Bay/281/2015(H7N3)
A/sanderling/Delaware_Bay/280/2015(H7N3)
A/ruddy_turnstone/New_Jersey/UGAI15-2958/2015(H7N3)
A/chicken/Texas/9686-3/2014(H6N5)
A/mallard/South_Dakota/AH0038067/2016(H7N7)
A/blue-winged_teal/SK/109_1595/2016(H7N6)
A/mallard/Maryland/11OS906/2011(H12N5)
A/blue-winged_teal/Missouri/12OS2402/2012(H4N8)
A/blue-winged_teal/North_Dakota/Sg-00707/2008(H4N8)
A/blue-winged_teal/Alberta/387/2007(H3N8)
A/blue-winged_Teal/Minnesota/AI09-3575/2009(H4N8)
A/blue-winged_teal/Guatemala/CIP049-05/2010(H3N8)
A/mallard/Alberta/590/2010(H7N8)
A/blue-winged_teal/Guatemala/CIP049-12/2010(H5N4)
A/mallard/California/2961/2011(H2N3)
A/northern_shoveler/California/2810/2011(H11N2)
A/northern_pintail/Nevada/AH0033473/2015(H3N8)
A/cinnamon_teal/Utah/AH0067154/2015(H7N7)
A/northern_shoveler/California/AH0055278/2015(H7N9)
A/Qingyuan/GIRD01/2017(H7N9)
A/Guangdong/HP001/2017(H7N9)
A/Shanghai/01/2014(H7N9)
A/chicken/Longquan/LQ78/2016(H7N9)
A/Beijing/1/2013(H7N9)
North American Lineage
H7N9/China
0.005

(E) PA

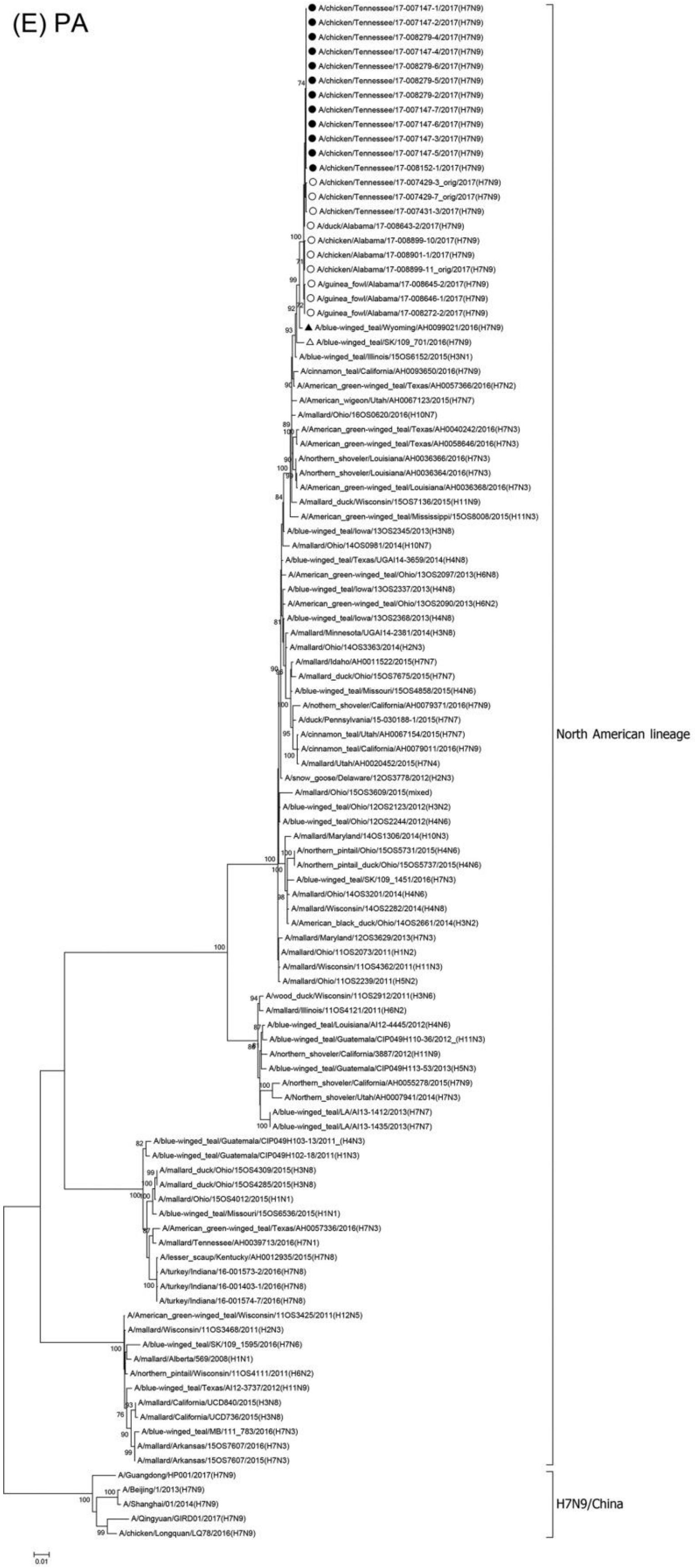

A/chicken/Tennessee/17-007147-1/2017(H7N9)
A/chicken/Tennessee/17-007147-2/2017(H7N9)
A/chicken/Tennessee/17-008279-4/2017(H7N9)
A/chicken/Tennessee/17-007147-4/2017(H7N9)
A/chicken/Tennessee/17-008279-6/2017(H7N9)
A/chicken/Tennessee/17-008279-5/2017(H7N9)
A/chicken/Tennessee/17-008279-2/2017(H7N9)
A/chicken/Tennessee/17-007147-7/2017(H7N9)
A/chicken/Tennessee/17-007147-6/2017(H7N9)
A/chicken/Tennessee/17-007147-3/2017(H7N9)
A/chicken/Tennessee/17-007147-5/2017(H7N9)
A/chicken/Tennessee/17-008152-1/2017(H7N9)
A/chicken/Tennessee/17-007429-3_orig/2017(H7N9)
A/chicken/Tennessee/17-007429-7_orig/2017(H7N9)
A/chicken/Tennessee/17-007431-3/2017(H7N9)
A/duck/Alabama/17-008643-2/2017(H7N9)
A/chicken/Alabama/17-008899-10/2017(H7N9)
A/chicken/Alabama/17-008901-1/2017(H7N9)
A/chicken/Alabama/17-008899-11_orig/2017(H7N9)
A/guinea_fowl/Alabama/17-008645-2/2017(H7N9)
A/guinea_fowl/Alabama/17-008646-1/2017(H7N9)
A/guinea_fowl/Alabama/17-008272-2/2017(H7N9)
A/blue-winged_teal/Wyoming/AH0099021/2016(H7N9)
A/blue-winged_teal/SK/109_701/2016(H7N9)
A/blue-winged_teal/Illinois/15OS6152/2015(H3N1)
A/cinnamon_teal/California/AH0093650/2016(H7N9)
A/American_green-winged_teal/Texas/AH0057366/2016(H7N2)
A/American_wigeon/Utah/AH0067123/2015(H7N7)
A/mallard/Ohio/16OS0620/2016(H10N7)
A/American_green-winged_teal/Texas/AH0040242/2016(H7N3)
A/American_green-winged_teal/Texas/AH0058646/2016(H7N3)
A/northern_shoveler/Louisiana/AH0036366/2016(H7N3)
A/northern_shoveler/Louisiana/AH0036364/2016(H7N3)
A/American_green-winged_teal/Louisiana/AH0036368/2016(H7N3)
A/mallard_duck/Wisconsin/15OS7136/2015(H11N9)
A/American_green-winged_teal/Mississippi/15OS8008/2015(H11N3)
A/blue-winged_teal/Iowa/13OS2345/2013(H3N8)
A/mallard/Ohio/14OS0981/2014(H10N7)
A/blue-winged_teal/Texas/UGAI14-3659/2014(H4N8)
A/American_green-winged_teal/Ohio/13OS2097/2013(H6N8)
A/blue-winged_teal/Iowa/13OS2337/2013(H4N8)
A/American_green-winged_teal/Ohio/13OS2090/2013(H6N2)
A/blue-winged_teal/Iowa/13OS2368/2013(H4N8)
A/mallard/Minnesota/UGAI14-2381/2014(H3N8)
A/mallard/Ohio/14OS3363/2014(H2N3)
A/mallard/Idaho/AH0011522/2015(H7N7)
A/mallard_duck/Ohio/15OS7675/2015(H7N7)
A/blue-winged_teal/Missouri/15OS4858/2015(H4N6)
A/nothern_shoveler/California/AH0079371/2016(H7N9)
A/duck/Pennsylvania/15-030188-1/2015(H7N7)
A/cinnamon_teal/Utah/AH0067154/2015(H7N7)
A/cinnamon_teal/California/AH0079011/2016(H7N9)
A/mallard/Utah/AH0020452/2015(H7N4)
A/snow_goose/Delaware/12OS3778/2012(H2N3)
A/mallard/Ohio/15OS3609/2015(mixed)
A/blue-winged_teal/Ohio/12OS2123/2012(H3N2)
A/blue-winged_teal/Ohio/12OS2244/2012(H4N6)
A/mallard/Maryland/14OS1306/2014(H10N3)
A/northern_pintail/Ohio/15OS5731/2015(H4N6)
A/northern_pintail_duck/Ohio/15OS5737/2015(H4N6)
A/blue-winged_teal/SK/109_1451/2016(H7N3)
A/mallard/Ohio/14OS3201/2014(H4N6)
A/mallard/Wisconsin/14OS2282/2014(H4N8)
A/American_black_duck/Ohio/14OS2661/2014(H3N2)
A/mallard/Maryland/12OS3629/2013(H7N3)
A/mallard/Ohio/11OS2073/2011(H1N2)
A/mallard/Wisconsin/11OS4362/2011(H11N3)
A/mallard/Ohio/11OS2239/2011(H5N2)
A/wood_duck/Wisconsin/11OS2912/2011(H3N6)
A/mallard/Illinois/11OS4121/2011(H6N2)
A/blue-winged_teal/Louisiana/AI12-4445/2012(H4N6)
A/blue-winged_teal/Guatemala/CIP049H110-36/2012_(H11N3)
A/northern_shoveler/California/3887/2012(H11N9)
A/blue-winged_teal/Guatemala/CIP049H113-53/2013(H5N3)
A/northern_shoveler/California/AH0055278/2015(H7N9)
A/Northern_shoveler/Utah/AH0007941/2014(H7N3)
A/blue-winged_teal/LA/AI13-1412/2013(H7N7)
A/blue-winged_teal/LA/AI13-1435/2013(H7N7)
A/blue-winged_teal/Guatemala/CIP049H103-13/2011_(H4N3)
A/blue-winged_teal/Guatemala/CIP049H102-18/2011(H1N3)
A/mallard_duck/Ohio/15OS4309/2015(H3N8)
A/mallard_duck/Ohio/15OS4285/2015(H3N8)
A/mallard/Ohio/15OS4012/2015(H1N1)
A/blue-winged_teal/Missouri/15OS6536/2015(H1N1)
A/American_green-winged_teal/Texas/AH0057336/2016(H7N3)
A/mallard/Tennessee/AH0039713/2016(H7N1)
A/lesser_scaup/Kentucky/AH0012935/2015(H7N8)
A/turkey/Indiana/16-001573-2/2016(H7N8)
A/turkey/Indiana/16-001403-1/2016(H7N8)
A/turkey/Indiana/16-001574-7/2016(H7N8)
A/American_green-winged_teal/Wisconsin/11OS3425/2011(H12N5)
A/mallard/Wisconsin/11OS3468/2011(H2N3)
A/blue-winged_teal/SK/109_1595/2016(H7N6)
A/mallard/Alberta/569/2008(H1N1)
A/northern_pintail/Wisconsin/11OS4111/2011(H6N2)
A/blue-winged_teal/Texas/AI12-3737/2012(H11N9)
A/mallard/California/UCD840/2015(H3N8)
A/mallard/California/UCD736/2015(H3N8)
A/blue-winged_teal/MB/111_783/2016(H7N3)
A/mallard/Arkansas/15OS7607/2016(H7N3)
A/mallard/Arkansas/15OS7607/2015(H7N3)
A/Guangdong/HP001/2017(H7N9)
A/Beijing/1/2013(H7N9)
A/Shanghai/01/2014(H7N9)
A/Qingyuan/GIRD01/2017(H7N9)
A/chicken/Longquan/LQ78/2016(H7N9)
North American lineage
H7N9/China
0.01

(F) NP

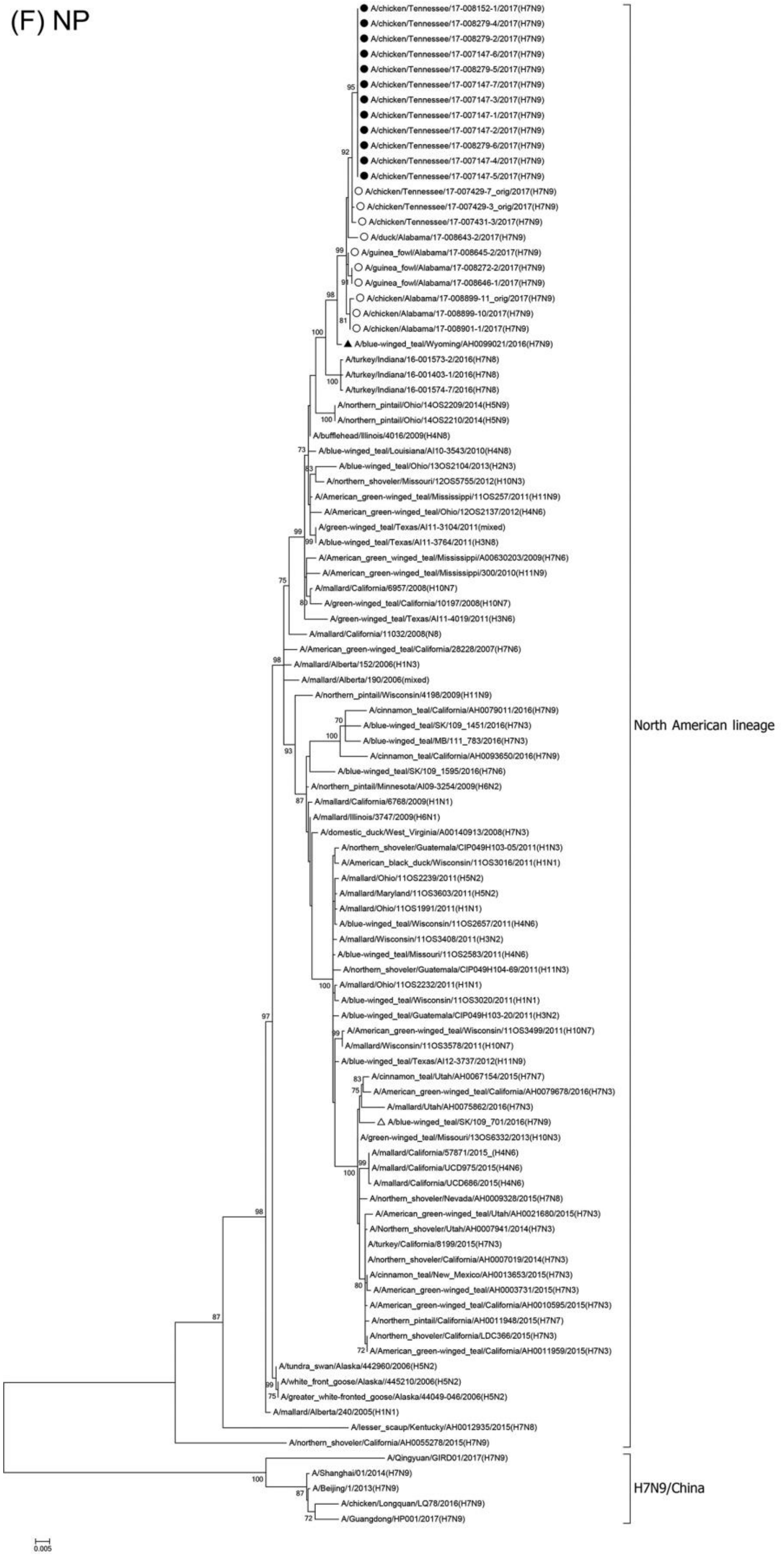
A/chicken/Tennessee/17-008152-1/2017(H7N9)
A/chicken/Tennessee/17-008279-4/2017(H7N9)
A/chicken/Tennessee/17-008279-2/2017(H7N9)
A/chicken/Tennessee/17-007147-6/2017(H7N9)
A/chicken/Tennessee/17-008279-5/2017(H7N9)
A/chicken/Tennessee/17-007147-7/2017(H7N9)
A/chicken/Tennessee/17-007147-3/2017(H7N9)
A/chicken/Tennessee/17-007147-1/2017(H7N9)
A/chicken/Tennessee/17-007147-2/2017(H7N9)
A/chicken/Tennessee/17-008279-6/2017(H7N9)
A/chicken/Tennessee/17-007147-4/2017(H7N9)
A/chicken/Tennessee/17-007147-5/2017(H7N9)
A/chicken/Tennessee/17-007429-7_orig/2017(H7N9)
A/chicken/Tennessee/17-007429-3_orig/2017(H7N9)
A/chicken/Tennessee/17-007431-3/2017(H7N9)
A/duck/Alabama/17-008643-2/2017(H7N9)
A/guinea_fowl/Alabama/17-008645-2/2017(H7N9)
A/guinea_fowl/Alabama/17-008272-2/2017(H7N9)
A/guinea_fowl/Alabama/17-008646-1/2017(H7N9)
A/chicken/Alabama/17-008899-11_orig/2017(H7N9)
A/chicken/Alabama/17-008899-10/2017(H7N9)
A/chicken/Alabama/17-008901-1/2017(H7N9)
A/blue-winged_teal/Wyoming/AH0099021/2016(H7N9)
A/turkey/Indiana/16-001573-2/2016(H7N8)
A/turkey/Indiana/16-001403-1/2016(H7N8)
A/turkey/Indiana/16-001574-7/2016(H7N8)
A/northern_pintail/Ohio/14OS2209/2014(H5N9)
A/northern_pintail/Ohio/14OS2210/2014(H5N9)
A/bufflehead/Illinois/4016/2009(H4N8)
A/blue-winged_teal/Louisiana/AI10-3543/2010(H4N8)
A/blue-winged_teal/Ohio/13OS2104/2013(H2N3)
A/northern_shoveler/Missouri/12OS5755/2012(H10N3)
A/American_green-winged_teal/Mississippi/11OS257/2011(H11N9)
A/American_green-winged_teal/Ohio/12OS2137/2012(H4N6)
A/green-winged_teal/Texas/AI11-3104/2011(mixed)
A/blue-winged_teal/Texas/AI11-3764/2011(H3N8)
A/American_green_winged_teal/Mississippi/A00630203/2009(H7N6)
A/American_green-winged_teal/Mississippi/300/2010(H11N9)
A/mallard/California/6957/2008(H10N7)
A/green-winged_teal/California/10197/2008(H10N7)
A/green-winged_teal/Texas/AI11-4019/2011(H3N6)
A/mallard/California/11032/2008(N8)
A/American_green-winged_teal/California/28228/2007(H7N6)
A/mallard/Alberta/152/2006(H1N3)
A/mallard/Alberta/190/2006(mixed)
A/northern_pintail/Wisconsin/4198/2009(H11N9)
A/cinnamon_teal/California/AH0079011/2016(H7N9)
A/blue-winged_teal/SK/109_1451/2016(H7N3)
A/blue-winged_teal/MB/111_783/2016(H7N3)
A/cinnamon_teal/California/AH0093650/2016(H7N9)
A/blue-winged_teal/SK/109_1595/2016(H7N6)
A/northern_pintail/Minnesota/AI09-3254/2009(H6N2)
A/mallard/California/6768/2009(H1N1)
A/mallard/Illinois/3747/2009(H6N1)
A/domestic_duck/West_Virginia/A00140913/2008(H7N3)
A/northern_shoveler/Guatemala/CIP049H103-05/2011(H1N3)
A/American_black_duck/Wisconsin/11OS3016/2011(H1N1)
A/mallard/Ohio/11OS2239/2011(H5N2)
A/mallard/Maryland/11OS3603/2011(H5N2)
A/mallard/Ohio/11OS1991/2011(H1N1)
A/blue-winged_teal/Wisconsin/11OS2657/2011(H4N6)
A/mallard/Wisconsin/11OS3408/2011(H3N2)
A/blue-winged_teal/Missouri/11OS2583/2011(H4N6)
A/northern_shoveler/Guatemala/CIP049H104-69/2011(H11N3)
A/mallard/Ohio/11OS2232/2011(H1N1)
A/blue-winged_teal/Wisconsin/11OS3020/2011(H1N1)
A/blue-winged_teal/Guatemala/CIP049H103-20/2011(H3N2)
A/American_green-winged_teal/Wisconsin/11OS3499/2011(H10N7)
A/mallard/Wisconsin/11OS3578/2011(H10N7)
A/blue-winged_teal/Texas/AI12-3737/2012(H11N9)
A/cinnamon_teal/Utah/AH0067154/2015(H7N7)
A/American_green-winged_teal/California/AH0079678/2016(H7N3)
A/mallard/Utah/AH0075862/2016(H7N3)
A/blue-winged_teal/SK/109_701/2016(H7N9)
A/green-winged_teal/Missouri/13OS6332/2013(H10N3)
A/mallard/California/57871/2015_(H4N6)
A/mallard/California/UCD975/2015(H4N6)
A/mallard/California/UCD686/2015(H4N6)
A/northern_shoveler/Nevada/AH0009328/2015(H7N8)
A/American_green-winged_teal/Utah/AH0021680/2015(H7N3)
A/Northern_shoveler/Utah/AH0007941/2014(H7N3)
A/turkey/California/8199/2015(H7N3)
A/northern_shoveler/California/AH0007019/2014(H7N3)
A/cinnamon_teal/New_Mexico/AH0013653/2015(H7N3)
A/American_green-winged_teal/AH0003731/2015(H7N3)
A/American_green-winged_teal/California/AH0010595/2015(H7N3)
A/northern_pintail/California/AH0011948/2015(H7N7)
A/northern_shoveler/California/LDC366/2015(H7N3)
A/American_green-winged_teal/California/AH0011959/2015(H7N3)
A/tundra_swan/Alaska/442960/2006(H5N2)
A/white_front_goose/Alaska//445210/2006(H5N2)
A/greater_white-fronted_goose/Alaska/44049-046/2006(H5N2)
A/mallard/Alberta/240/2005(H1N1)
A/lesser_scaup/Kentucky/AH0012935/2015(H7N8)
A/northern_shoveler/California/AH0055278/2015(H7N9)
A/Qingyuan/GIRD01/2017(H7N9)
A/Shanghai/01/2014(H7N9)
A/Beijing/1/2013(H7N9)
A/chicken/Longquan/LQ78/2016(H7N9)
A/Guangdong/HP001/2017(H7N9)
North American lineage
H7N9/China
0.005

(G) M

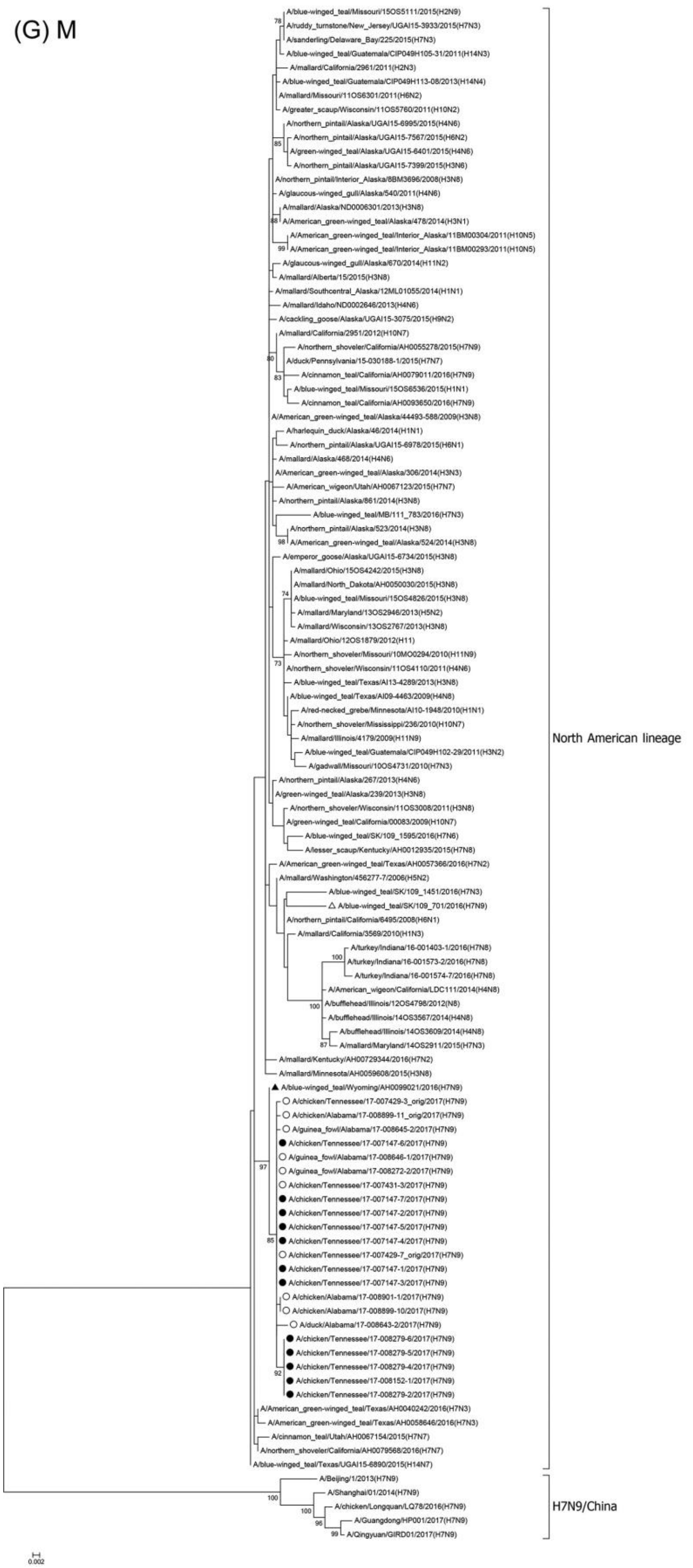

A/blue-winged_teal/Missouri/15OS5111/2015(H2N9)
A/ruddy_turnstone/New_Jersey/UGAI15-3933/2015(H7N3)
A/sanderling/Delaware_Bay/225/2015(H7N3)
A/blue-winged_teal/Guatemala/CIP049H105-31/2011(H14N3)
A/mallard/California/2961/2011(H2N3)
A/blue-winged_teal/Guatemala/CIP049H113-08/2013(H14N4)
A/mallard/Missouri/11OS6301/2011(H6N2)
A/greater_scaup/Wisconsin/11OS5760/2011(H10N2)
A/northern_pintail/Alaska/UGAI15-6995/2015(H4N6)
A/northern_pintail/Alaska/UGAI15-7567/2015(H6N2)
A/green-winged_teal/Alaska/UGAI15-6401/2015(H4N6)
A/northern_pintail/Alaska/UGAI15-7399/2015(H3N6)
A/northern_pintail/Interior_Alaska/8BM3696/2008(H3N8)
A/glaucous-winged_gull/Alaska/540/2011(H4N6)
A/mallard/Alaska/ND0006301/2013(H3N8)
A/American_green-winged_teal/Alaska/478/2014(H3N1)
A/American_green-winged_teal/Interior_Alaska/11BM00304/2011(H10N5)
A/American_green-winged_teal/Interior_Alaska/11BM00293/2011(H10N5)
A/glaucous-winged_gull/Alaska/670/2014(H11N2)
A/mallard/Alberta/15/2015(H3N8)
A/mallard/Southcentral_Alaska/12ML01055/2014(H1N1)
A/mallard/Idaho/ND0002646/2013(H4N6)
A/cackling_goose/Alaska/UGAI15-3075/2015(H9N2)
A/mallard/California/2951/2012(H10N7)
A/northern_shoveler/California/AH0055278/2015(H7N9)
A/duck/Pennsylvania/15-030188-1/2015(H7N7)
A/cinnamon_teal/California/AH0079011/2016(H7N9)
A/blue-winged_teal/Missouri/15OS6536/2015(H1N1)
A/cinnamon_teal/California/AH0093650/2016(H7N9)
A/American_green-winged_teal/Alaska/44493-588/2009(H3N8)
A/harlequin_duck/Alaska/46/2014(H1N1)
A/northern_pintail/Alaska/UGAI15-6978/2015(H6N1)
A/mallard/Alaska/468/2014(H4N6)
A/American_green-winged_teal/Alaska/306/2014(H3N3)
A/American_wigeon/Utah/AH0067123/2015(H7N7)
A/northern_pintail/Alaska/861/2014(H3N8)
A/blue-winged_teal/MB/111_783/2016(H7N3)
A/northern_pintail/Alaska/523/2014(H3N8)
A/American_green-winged_teal/Alaska/524/2014(H3N8)
A/emperor_goose/Alaska/UGAI15-6734/2015(H3N8)
A/mallard/Ohio/15OS4242/2015(H3N8)
A/mallard/North_Dakota/AH0050030/2015(H3N8)
A/blue-winged_teal/Missouri/15OS4826/2015(H3N8)
A/mallard/Maryland/13OS2946/2013(H5N2)
A/mallard/Wisconsin/13OS2767/2013(H3N8)
A/mallard/Ohio/12OS1879/2012(H11)
A/northern_shoveler/Missouri/10MO0294/2010(H11N9)
A/northern_shoveler/Wisconsin/11OS4110/2011(H4N6)
A/blue-winged_teal/Texas/AI13-4289/2013(H3N8)
A/blue-winged_teal/Texas/AI09-4463/2009(H4N8)
A/red-necked_grebe/Minnesota/AI10-1948/2010(H1N1)
A/northern_shoveler/Mississippi/236/2010(H10N7)
A/mallard/Illinois/4179/2009(H11N9)
A/blue-winged_teal/Guatemala/CIP049H102-29/2011(H3N2)
A/gadwall/Missouri/10OS4731/2010(H7N3)
North American lineage
A/northern_pintail/Alaska/267/2013(H4N6)
A/green-winged_teal/Alaska/239/2013(H3N8)
A/northern_shoveler/Wisconsin/11OS3008/2011(H3N8)
A/green-winged_teal/California/00083/2009(H10N7)
A/blue-winged_teal/SK/109_1595/2016(H7N6)
A/lesser_scaup/Kentucky/AH0012935/2015(H7N8)
A/American_green-winged_teal/Texas/AH0057366/2016(H7N2)
A/mallard/Washington/456277-7/2006(H5N2)
A/blue-winged_teal/SK/109_1451/2016(H7N3)
A/blue-winged_teal/SK/109_701/2016(H7N9)
A/northern_pintail/California/6495/2008(H6N1)
A/mallard/California/3569/2010(H1N3)
A/turkey/Indiana/16-001403-1/2016(H7N8)
A/turkey/Indiana/16-001573-2/2016(H7N8)
A/turkey/Indiana/16-001574-7/2016(H7N8)
A/American_wigeon/California/LDC111/2014(H4N8)
A/bufflehead/Illinois/12OS4798/2012(N8)
A/bufflehead/Illinois/14OS3567/2014(H4N8)
A/bufflehead/Illinois/14OS3609/2014(H4N8)
A/mallard/Maryland/14OS2911/2015(H7N3)
A/mallard/Kentucky/AH00729344/2016(H7N2)
A/mallard/Minnesota/AH0059608/2015(H3N8)
A/blue-winged_teal/Wyoming/AH0099021/2016(H7N9)
A/chicken/Tennessee/17-007429-3_orig/2017(H7N9)
A/chicken/Alabama/17-008899-11_orig/2017(H7N9)
A/guinea_fowl/Alabama/17-008645-2/2017(H7N9)
A/chicken/Tennessee/17-007147-6/2017(H7N9)
A/guinea_fowl/Alabama/17-008646-1/2017(H7N9)
A/guinea_fowl/Alabama/17-008272-2/2017(H7N9)
A/chicken/Tennessee/17-007431-3/2017(H7N9)
A/chicken/Tennessee/17-007147-7/2017(H7N9)
A/chicken/Tennessee/17-007147-2/2017(H7N9)
A/chicken/Tennessee/17-007147-5/2017(H7N9)
A/chicken/Tennessee/17-007147-4/2017(H7N9)
A/chicken/Tennessee/17-007429-7_orig/2017(H7N9)
A/chicken/Tennessee/17-007147-1/2017(H7N9)
A/chicken/Tennessee/17-007147-3/2017(H7N9)
A/chicken/Alabama/17-008901-1/2017(H7N9)
A/chicken/Alabama/17-008899-10/2017(H7N9)
A/duck/Alabama/17-008643-2/2017(H7N9)
A/chicken/Tennessee/17-008279-6/2017(H7N9)
A/chicken/Tennessee/17-008279-5/2017(H7N9)
A/chicken/Tennessee/17-008279-4/2017(H7N9)
A/chicken/Tennessee/17-008152-1/2017(H7N9)
A/chicken/Tennessee/17-008279-2/2017(H7N9)
A/American_green-winged_teal/Texas/AH0040242/2016(H7N3)
A/American_green-winged_teal/Texas/AH0058646/2016(H7N3)
A/cinnamon_teal/Utah/AH0067154/2015(H7N7)
A/northern_shoveler/California/AH0079568/2016(H7N7)
A/blue-winged_teal/Texas/UGAI15-6890/2015(H14N7)
A/Beijing/1/2013(H7N9)
A/Shanghai/01/2014(H7N9)
A/chicken/Longquan/LQ78/2016(H7N9)
A/Guangdong/HP001/2017(H7N9)
A/Qingyuan/GIRD01/2017(H7N9)
H7N9/China
0.002

(H) NS

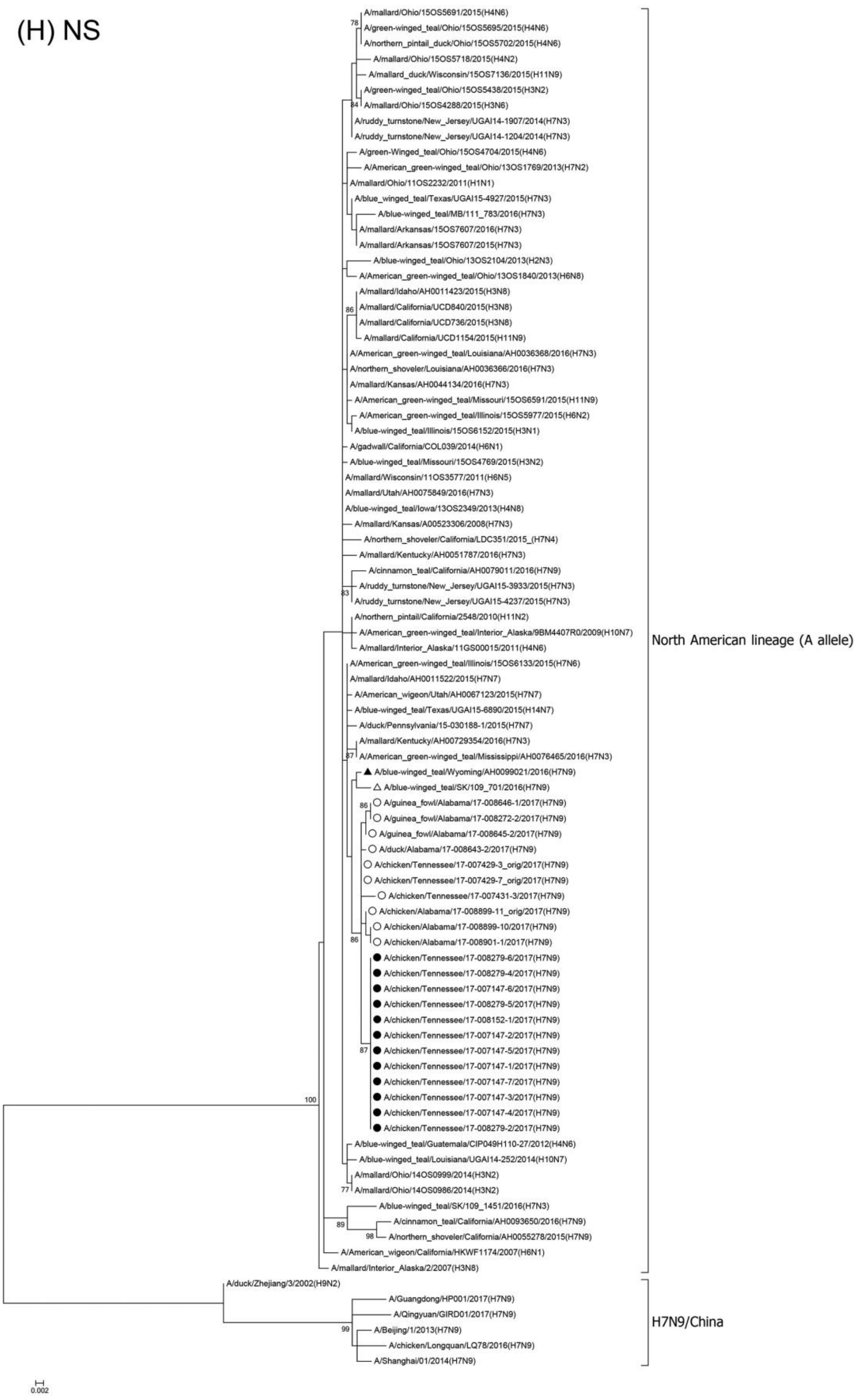

A/mallard/Ohio/15OS5691/2015(H4N6)
78
A/green-winged_teal/Ohio/15OS5695/2015(H4N6)
A/northern_pintail_duck/Ohio/15OS5702/2015(H4N6)
A/mallard/Ohio/15OS5718/2015(H4N2)
A/mallard_duck/Wisconsin/15OS7136/2015(H11N9)
A/green-winged_teal/Ohio/15OS5438/2015(H3N2)
84
A/mallard/Ohio/15OS4288/2015(H3N6)
A/ruddy_turnstone/New_Jersey/UGAI14-1907/2014(H7N3)
A/ruddy_turnstone/New_Jersey/UGAI14-1204/2014(H7N3)
A/green-Winged_teal/Ohio/15OS4704/2015(H4N6)
A/American_green-winged_teal/Ohio/13OS1769/2013(H7N2)
A/mallard/Ohio/11OS2232/2011(H1N1)
A/blue_winged_teal/Texas/UGAI15-4927/2015(H7N3)
A/blue-winged_teal/MB/111_783/2016(H7N3)
A/mallard/Arkansas/15OS7607/2016(H7N3)
A/mallard/Arkansas/15OS7607/2015(H7N3)
A/blue-winged_teal/Ohio/13OS2104/2013(H2N3)
A/American_green-winged_teal/Ohio/13OS1840/2013(H6N8)
A/mallard/Idaho/AH0011423/2015(H3N8)
86
A/mallard/California/UCD840/2015(H3N8)
A/mallard/California/UCD736/2015(H3N8)
A/mallard/California/UCD1154/2015(H11N9)
A/American_green-winged_teal/Louisiana/AH0036368/2016(H7N3)
A/northern_shoveler/Louisiana/AH0036366/2016(H7N3)
A/mallard/Kansas/AH0044134/2016(H7N3)
A/American_green-winged_teal/Missouri/15OS6591/2015(H11N9)
A/American_green-winged_teal/Illinois/15OS5977/2015(H6N2)
A/blue-winged_teal/Illinois/15OS6152/2015(H3N1)
A/gadwall/California/COL039/2014(H6N1)
A/blue-winged_teal/Missouri/15OS4769/2015(H3N2)
A/mallard/Wisconsin/11OS3577/2011(H6N5)
A/mallard/Utah/AH0075849/2016(H7N3)
A/blue-winged_teal/Iowa/13OS2349/2013(H4N8)
A/mallard/Kansas/A00523306/2008(H7N3)
A/northern_shoveler/California/LDC351/2015_(H7N4)
A/mallard/Kentucky/AH0051787/2016(H7N3)
A/cinnamon_teal/California/AH0079011/2016(H7N9)
A/ruddy_turnstone/New_Jersey/UGAI15-3933/2015(H7N3)
83
A/ruddy_turnstone/New_Jersey/UGAI15-4237/2015(H7N3)
A/northern_pintail/California/2548/2010(H11N2)
A/American_green-winged_teal/Interior_Alaska/9BM4407R0/2009(H10N7)
A/mallard/Interior_Alaska/11GS00015/2011(H4N6)
A/American_green-winged_teal/Illinois/15OS6133/2015(H7N6)
A/mallard/Idaho/AH0011522/2015(H7N7)
A/American_wigeon/Utah/AH0067123/2015(H7N7)
A/blue-winged_teal/Texas/UGAI15-6890/2015(H14N7)
A/duck/Pennsylvania/15-030188-1/2015(H7N7)
A/mallard/Kentucky/AH00729354/2016(H7N3)
87
A/American_green-winged_teal/Mississippi/AH0076465/2016(H7N3)
▲ A/blue-winged_teal/Wyoming/AH0099021/2016(H7N9)
△ A/blue-winged_teal/SK/109_701/2016(H7N9)
86
○ A/guinea_fowl/Alabama/17-008646-1/2017(H7N9)
○ A/guinea_fowl/Alabama/17-008272-2/2017(H7N9)
○ A/guinea_fowl/Alabama/17-008645-2/2017(H7N9)
○ A/duck/Alabama/17-008643-2/2017(H7N9)
○ A/chicken/Tennessee/17-007429-3_orig/2017(H7N9)
○ A/chicken/Tennessee/17-007429-7_orig/2017(H7N9)
○ A/chicken/Tennessee/17-007431-3/2017(H7N9)
○ A/chicken/Alabama/17-008899-11_orig/2017(H7N9)
○ A/chicken/Alabama/17-008899-10/2017(H7N9)
86
○ A/chicken/Alabama/17-008901-1/2017(H7N9)
● A/chicken/Tennessee/17-008279-6/2017(H7N9)
● A/chicken/Tennessee/17-008279-4/2017(H7N9)
● A/chicken/Tennessee/17-007147-6/2017(H7N9)
● A/chicken/Tennessee/17-008279-5/2017(H7N9)
● A/chicken/Tennessee/17-008152-1/2017(H7N9)
● A/chicken/Tennessee/17-007147-2/2017(H7N9)
87
● A/chicken/Tennessee/17-007147-5/2017(H7N9)
● A/chicken/Tennessee/17-007147-1/2017(H7N9)
● A/chicken/Tennessee/17-007147-7/2017(H7N9)
100
● A/chicken/Tennessee/17-007147-3/2017(H7N9)
● A/chicken/Tennessee/17-007147-4/2017(H7N9)
● A/chicken/Tennessee/17-008279-2/2017(H7N9)
A/blue-winged_teal/Guatemala/CIP049H110-27/2012(H4N6)
A/blue-winged_teal/Louisiana/UGAI14-252/2014(H10N7)
A/mallard/Ohio/14OS0999/2014(H3N2)
77
A/mallard/Ohio/14OS0986/2014(H3N2)
A/blue-winged_teal/SK/109_1451/2016(H7N3)
89
A/cinnamon_teal/California/AH0093650/2016(H7N9)
98
A/northern_shoveler/California/AH0055278/2015(H7N9)
A/American_wigeon/California/HKWF1174/2007(H6N1)
A/mallard/Interior_Alaska/2/2007(H3N8)
North American lineage (A allele)
A/duck/Zhejiang/3/2002(H9N2)
A/Guangdong/HP001/2017(H7N9)
A/Qingyuan/GIRD01/2017(H7N9)
99
A/Beijing/1/2013(H7N9)
A/chicken/Longquan/LQ78/2016(H7N9)
A/Shanghai/01/2014(H7N9)
H7N9/China
0.002

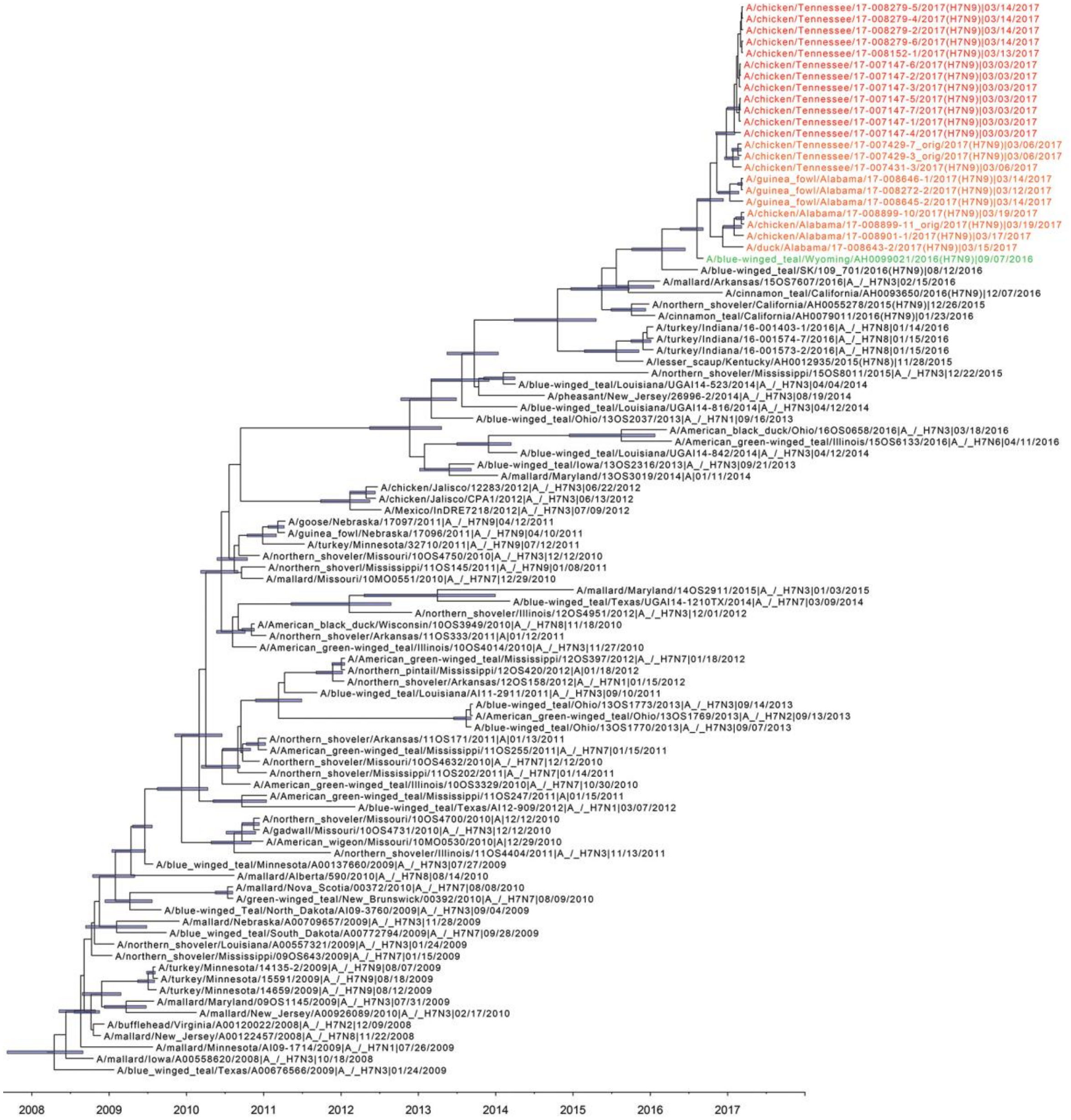

Technical Appendix Figure 3. Relaxed clock phylogenetic tree of H7N9 virus hemagglutinin gene. The phylogenetic relationships and temporal evolutionary history have been estimated by molecular clock analysis. Highly pathogenic avian influenza isolates (red), low pathogenicity avian influenza virus isolates (orange), and A/blue-winged teal/Wyoming/AH0099021/2016 (green) are indicated.

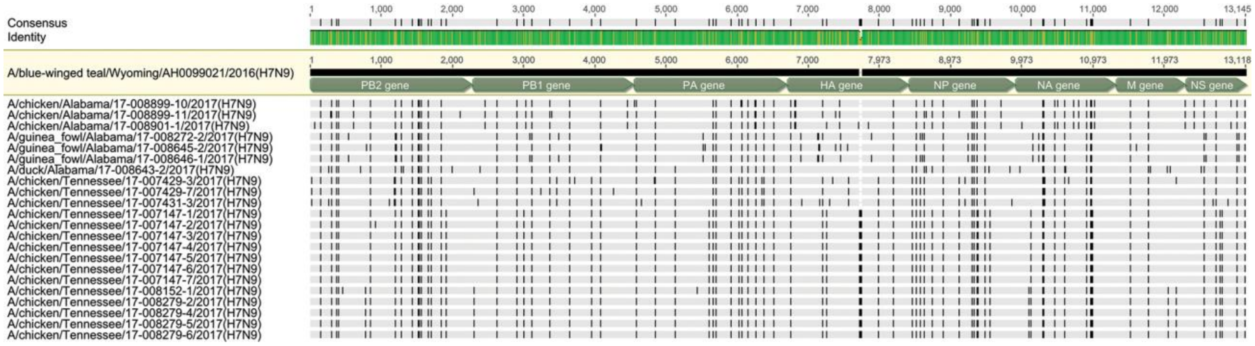

Technical Appendix Figure 4. Nucleotide sequence alignment of complete genome sequences of H7N9 viruses, United States, 2017. Alignment of the complete genome sequences was performed using MAFFT (http://mafft.cbrc.jp/alignment/software/). The complete genome sequence of low pathogenicity avian influenza virus A/blue-winged teal/Wyoming/AH0099021/2016(H7N9) was placed on top of the alignment and served as the reference. Vertical lines indicate nucleotide differences from the reference sequence. HA, hemagglutinin; M, matrix; NA, neuraminidase; NP, nucleoprotein; NS, nonstructural; PA, polymerase acidic; PB1, polymerase basic 1; PB2, polymerase basic 2.
